# Vorticity‐Facilitated Platelet Aggregation: a High Expansion‐Ratio Stenotic Microfluidic Platform Unravels the Role of Complex Flow Dynamics in Arterial Thrombosis

**DOI:** 10.1002/adhm.202500436

**Published:** 2025-06-04

**Authors:** Jianfang Ren, Nurul Aisha Zainal Abidin, Allan Sun, Rui Gao, Yuxin Chen, Arian Nasser, Zihao Wang, Yunduo Charles Zhao, Alexander Dupuy, Anna Waterhouse, Qian Peter Su, Daniele Vigolo, Mike Chia Lun Wu, Lining Arnold Ju

**Affiliations:** ^1^ School of Biomedical Engineering, Faculty of Engineering The University of Sydney Darlington NSW 2008 Australia; ^2^ Charles Perkins Centre The University of Sydney Camperdown NSW 2006 Australia; ^3^ The University of Sydney Nano Institute (Sydney Nano) The University of Sydney Camperdown NSW 2006 Australia; ^4^ Heart Research Institute Newtown NSW 2042 Australia; ^5^ School of Medical Sciences Faculty Medicine and Health The University of Sydney Camperdown NSW 2006 Australia; ^6^ School of Biomedical Engineering The University of Technology Sydney Ultimo NSW 2007 Australia

**Keywords:** arterial thrombosis, mechanobiology, microfluidic, platelet aggregation, stenosis, vorticity

## Abstract

Complex flow patterns play a critical role in arterial thrombosis, yet the specific contribution of vorticity—the rotational component of fluid flow—remains poorly understood. An innovative microfluidic platform with systematically varied expansion angles (β = 30°‐150°) in a double stenosis design is developed to isolate vorticity's effects under controlled conditions. The high expansion‐ratio device with sharp‐angled geometries successfully generates distinct vortical flow patterns, confirmed through computational and experimental flow visualizations. Real‐time confocal microscopy revealed a strong positive correlation (*r* = 0.6698) between vorticity magnitude and thrombus size, with high‐vorticity conditions producing thrombi up to four times larger than low‐vorticity settings. Mechanistic investigations demonstrated enhanced von Willebrand Factor (vWF) accumulation and platelet integrin activation in vortical environments. Platelets in high‐vorticity regions exhibited integrin α_IIb_β_3_ intermediate activation states with significantly enhanced calcium signaling, suggesting vorticity amplifies platelet mechanosensing pathways. Inhibition of the interaction between vWF and platelet glycoprotein Ibα (GPIbα) receptor abolished biomechanical platelet aggregation in vortical regions. These findings provide valuable insights into platelet thrombosis in complex flow environments with significant implications for optimizing medical devices to minimize thrombotic complications associated with vortex formation.

## Graphical abstract

This study reveals how vorticity—a swirling flow pattern—enhances platelet aggregation and thrombus formation in high‐shear conditions. Using a custom microfluidic model, it is shows that vorticity promotes vWF–GPIbα interactions and calcium signaling, providing new insights into thrombosis mechanobiology and informing safer blood‐contacting medical device designs.

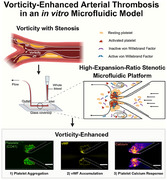

## 1 Introduction

Arterial thrombosis, characterized by the excessive formation of platelet rich blood clots within arteries, remains a leading cause of acute coronary syndrome, myocardial infarction, and stroke worldwide.^[^ ^66^, ^75^ ^]^ Despite advancements in medical technology, the complex interplay between hemostasis and thrombosis, coupled with the challenge of optimizing antithrombotic interventions, continues to pose significant clinical hurdles.^[^ ^67^, ^68^ ^]^ While Virchow's Triad has long identified hemodynamic alterations, endothelial dysfunction, and hypercoagulability as critical drivers of thrombosis,^[^ ^1^, ^74^ ^]^ our study focuses specifically on the hemodynamic component, particularly shear‐induced platelet aggregation (SIPA) and the understudied role of vorticity in this process.^[^ ^2^ ^]^ SIPA is especially relevant to arterial thrombosis, producing platelet‐rich thrombi under conditions of elevated wall shear rates exceeding 1,000 s⁻¹.^[^ ^3^ ^]^ These high shear hemodynamic conditions profoundly affect platelet behavior and aggregation through biomechanical mechanisms distinct from those involved in venous thrombosis.^[^ ^4^ ^]^ While considerable research has focused on the effects of flow velocity, wall shear rates, and shear gradients on platelet function, the impact of vorticity—a measure of local fluid rotation—remains largely unexplored in the context of thrombosis mechanobiology. Vorticity is particularly relevant in steep stenotic geometries, which are common in sudden vessel narrowing, such as atherosclerotic plaques, and at the interfaces of implanted medical devices.^[^ ^5^ ^]^ Understanding the role of vorticity in platelet aggregation will provide crucial insights into thrombosis risk in complex flow environments and inform the design of safer medical devices and more effective antithrombotic strategies.

The biomechanical aggregation of platelets in high‐shear environments is primarily mediated by the interaction between von Willebrand Factor (vWF) and the platelet receptor glycoprotein Ibα (GPIbα).^[^ ^6^ ^]^ Under high shear conditions, vWF undergoes conformational changes that expose binding sites for GPIbα, initiating platelet adhesion and activation.^[^ ^7^ ^]^ Subsequently, activation of the platelet integrin α_IIb_β_3_ leads to firm adhesion and platelet‐platelet interactions.^[^ ^8^ ^]^ While these mechanosensing mechanisms have been well‐characterized in the context of uniform shear flows, their behavior in complex, vortical flow fields remains poorly understood. The rotational fluid motion characteristic of vortices may induce unique conformational changes in vWF or alter the dynamics of platelet–vWF interactions in ways that are not observed in linear flow fields.^[^ ^9^, ^10^ ^]^ Understanding vorticity's impact on platelet mechanosensing is crucial for developing a comprehensive understanding of thrombosis in pathophysiologically relevant flow conditions.

Microfluidic platforms have emerged as powerful tools for investigating SIPA in arterial thrombosis, offering flexibility in design, rapid turnaround for results, and reduced sample requirements compared to traditional flow chambers.^[^ ^2^, ^11^ ^]^ These advantages make microfluidic devices particularly suitable for developing point‐of‐care diagnostics and for high‐throughput screening of antithrombotic therapies, as demonstrated by several research groups.^[^ ^2^, ^12^, ^13^ ^]^ Among the various microfluidic configurations, stenotic designs have proven especially valuable for studying complex hemodynamic parameters, including vorticity, recirculation, and shear gradients.^[^ ^14^, ^15^ ^]^ By modulating the angles of stenosis, researchers can produce a range of flow profiles, with laminar and disturbed flow, that mimic physiological and pathological conditions. Stenotic microfluidics have revealed the biphasic nature of SIPA, with initial platelet recruitment occurring at the stenosis apex under high shear conditions, followed by aggregate growth in the low‐shear recirculation zone immediately downstream.^[^ ^16^ ^]^ These studies have emphasized the significance of shear gradients in promoting thrombus formation, even when bulk shear rates are similar to those in straight channel designs.^[^ ^17^ ^]^

**Figure 1:**
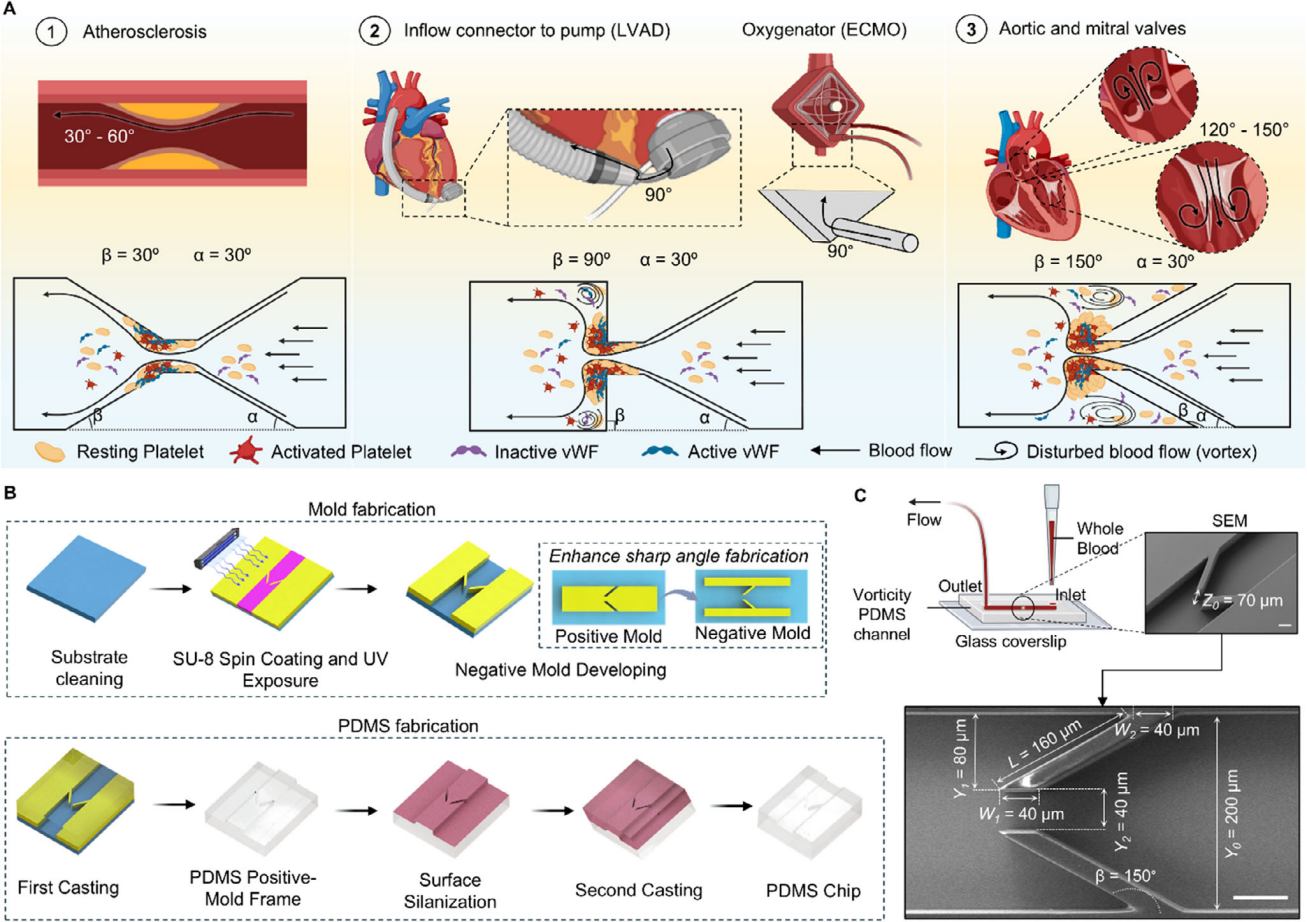
Device design and fabrication process of the vorticity‐channel model. A) Schematic showing complex flow patterns in cardiovascular conditions and medical devices contributing to thrombosis. Top row: blood flow through atherosclerotic plaques at angles of 30°–60° (1), connecting parts in LVAD and oxygenator in ECMO at 90° angles (2), aortic and mitral valves under pathological conditions (i.e., valve dysfunction and atrial fibrillation) (3). Bottom row: vorticity microfluidic channels with varying micro‐expansion geometries (expansion β angle): 30° (1), 90° (2), and 150° (3), all featuring a consistent contraction angle of 30° (contraction α angle). B) Double‐casting fabrication process of a high‐expansion‐angle (β = 150°) microfluidic channel. Unlike conventional mold fabrication that relies on creating a positive mold, we utilized UV lithography on SU‐8 photoresist laminate to produce a negative mold. This approach enabled a streamlined double‐casting process for the fabrication of high‐expansion‐ratio PDMS microchannels. C) Schematic of whole blood perfusion platform and SEM images of high‐expansion‐angle (β = 150°) vorticity microfluidic channel, featuring *Z*_*0*_ = 70 µm height and 80% reduction (with constriction width *W*_*1*_ = 40 µm, expansion width *W*_*2*_ = 40 µm and expansion length *L* = 160 µm) of *Y*_*0*_ = 200 µm channel inlet width in the stenosis region. The ratio *W*_*2*_/*L* represents the expansion ratio of the microchannel, which quantifies the degree of widening in the expansion zone relative to the channel width and presents a high‐aspects ratio of constriction zone. Scale bars: 50 µm. Illustration figures are created in https://BioRender.com and SolidWorks.

In this study, unlike previous studies that primarily focused on linear shear flows,^[^ ^18^ ^]^ we developed a novel microfluidic platform that allows for investigation of vortex effects on platelet activation and aggregation. Our design was inspired by the various geometries found in both pathological blood vessels and medical devices associated with thrombotic complications, such as atherosclerosis,^[^ ^19^ ^]^ Left Ventricular Assist Device (LVAD),^[^ ^2^ ^]^ Extracorporeal membrane oxygenation (ECMO),^[^ ^20^ ^]^ and aortic and mitral valves^[^ ^21^ ^]^ (**Figure** **1**A). Building upon previous studies that have investigated the effects of various hemodynamic parameters on platelet function,^[^ ^11^, ^22^, ^23^ ^]^ our work specifically focuses on examining and characterizing the effects of vorticity, which has received comparatively less attention in thrombosis research. We introduced a double stenosis structure within our microfluidic channels, defined by two key angles: α and β. The angle α represents the inclination of the front face of the stenotic hump relative to the incident flow direction, while β describes the angle of the back face where flow expansion occurs, controlling the dynamics of vortex formation (Figure 1A; Figure S1A, Supporting Information). This approach enables us to investigate the influence of vortices on platelet aggregation in addition to laminar flow, providing valuable insights into the mechanobiology of thrombosis. Our findings have significant implications for assessing thrombosis risk and designing hemocompatible medical devices.

## 2 Results

### 2.1 Design Rationale for Variable Angled Double Stenotic Microfluidics

The α angle was fixed at 30° for all designs, as this angle reasonably approximates the gradual narrowing often seen in stenotic vessels. The key feature of our approach lies in the systematic modulation of the β angle, ranging from 30° to 150°, to generate varying vorticity levels that replicate the complex flow patterns observed in cardiovascular conditions and blood‐contacting medical devices. This design strategy allowed us to emulate a range of clinically relevant scenarios (Figure 1A; Figure S1A, Supporting Information):

1. Low β angles (30° to 60°): These geometries mimic the flow conditions in narrowed vessels with atherosclerotic plaques, where gradual expansion occurs post‐constriction.
2. Intermediate β angles (90°): This design approximates the abrupt changes in flow patterns often encountered at the interface or junctions of medical devices such as ECMO circuits and LVADs.
3. High β angles (120° to 150°): These extreme expansion angles simulate the complex flow patterns observed in specific vascular geometries, such as those found around aortic and mitral valves. Clinically, pathological conditions like valve stenosis and regurgitation can result in disturbed flow vortices flow associated with increased thrombosis risk.^[^ ^24^ ^]^

By systematically varying the β angle while maintaining a constant α angle, we created a versatile platform capable of investigating a wide range of vorticity‐induced flow conditions and their effects on platelet aggregation and thrombus formation.

### 2.2 Microfabrication of High Expansion‐Ratio and Sharp‐Angled Stenotic Microfluidics

The fabrication of our microfluidic devices, particularly those with high β angles, presented significant challenges due to the high expansion ratio (aspect ratio of constriction region = 4:1 to 5:1) and sharp incline angles required. Traditional soft lithography techniques often struggle to accurately reproduce such complex geometries, while 3D printing fails to provide smooth surfaces and precise geometrical feature replication, especially the sharp corners and high‐aspect constriction in β = 150°.^[^ ^25^ ^]^

To overcome these challenges, we applied the double casting method, which was previously established by Guocheng et al., 2012 (Figure 1B).^[^ ^26^ ^]^ In this approach, we reversed the microfluidic channel pattern during the photolithography process to enhance development, enabling the formation of sharp‐angled structures. It involved creating a negative mold using SU8 photoresist on a silicon wafer, followed by forming a PDMS positive‐mold frame that underwent critical silanization surface treatment. The efficacy of our silanization process was confirmed through contact angle measurements (Figure S1B, Supporting Information), which demonstrated a significant change in the wettability of the PDMS surface. We focused on optimizing the silanization process and demolding techniques to achieve the high‐fidelity replication of sharp corners and smooth surfaces even in the challenging β = 150° design, building upon the foundation established by previous studies.^[^ ^27^, ^28^, ^29^ ^]^

The resulting microfluidic channels precisely replicated the designed geometries, featuring a height of 70 µm and an 80% reduction in width within the stenotic region, where the post‐constriction expansion ratio varies based on an initial inlet channel width of 200 µm (Figure 1C; Figure S1A, Supporting Information). Scanning Electron Microscopy (SEM) images of the fabricated devices revealed the high fidelity of our fabrication process, showcasing sharp corners and smooth surfaces even in high‐angle designs. (Figure S1B, Supporting Information)

The double‐casting method has been established for fabricating high‐aspect‐ratio microstructures by several research groups.^[^ ^30^, ^31^, ^32^ ^]^ This approach continues to demonstrate a strong capability in achieving complex soft‐base microgeometry or surface modification. For example, recent work by Margarida et al used this method to separate blood plasma.^[^ ^32^ ^]^ Our application of this established technique enabled the precise and reproducible creation of complex geometries that closely mimic pathological flow.^[^ ^32^, ^33^, ^34^ ^]^ The reliability of this approach was essential for accurately capturing vortex formations in sharp‐angle designs and ensuring comparable experimental flow conditions.

### 2.3 Hemodynamic Characterization of Vorticity‐Generating Microfluidic Geometries

To understand the flow dynamics within our microfluidic devices, we employed a dual flow visualization technique, comprising computational fluid dynamics (CFD) simulations and Ghost Particle Velocimetry (GPV) experiments. This comprehensive characterization was essential to establish the relationship between our geometric designs and the resulting flow patterns, particularly the formation of varying magnitudes of vortices. We simulated flow conditions corresponding to input shear rates from *γ*_*0*_ = 1,000 to 5,000 s^−1^, encompassing a range of physiologically and pathologically relevant flow conditions.^[^ ^2^, ^35^, ^36^ ^]^

**Figure 2:**
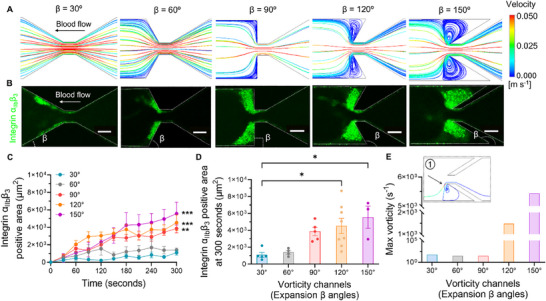
Hemodynamic characterization of vorticity‐generating microfluidic geometries. A) CFD simulated velocity streamlines at the mid‐plane of the microfluidic channels. B) Representative confocal images of platelet aggregation (anti‐integrin α_IIb_β_3_ mAb P2, *green*) in all geometries after 300 s of whole blood perfusion. The edge of the channels were marked with a white dotted line. Scale bars: 50 µm. C) Time course of thrombus size within different geometries. D) Thrombus size of different geometries at the endpoint (300 s). E) The arrow indicates the maximum *x*‐axis vorticity along a representative streamline, located above the stenosis apex spanning the shear acceleration and deceleration zones— regions where frictional forces are greatest (1)—at the mid‐plane (*z*‐axis) of the microfluidic channel. All the data were shown at *γ_0_* = 2,000 s^−1^ input shear rate. Channel edges are marked with white dotted lines. Data are presented as mean ± s.e.m., n ≥ 3; Statistical analysis: Kruskal‐Wallis with comparisons to 30° (C), One‐Way ANOVA with Tukey's post‐test (D). ^*^ *p* ≤ 0.05, ^**^ *p* ≤ 0.01, ^***^ *p* ≤ 0.001.

The CFD results revealed distinct flow patterns for each β angle, with pronounced vortex formation in the expansion zones of the higher‐angle designs (**Figure** **2**A; Figure S2A,B, Supporting Information). In β = 30°, the flow remained largely attached to the channel walls with minimal recirculation. As the β angle increased, we observed the development of intensifying vortices in the expansion zone from β = 30° to 150°. The β = 150° exhibited the most dramatic vortex formation, with a large recirculation zone occupying a significant portion of the post‐constriction area. (Figure 2A)

To validate these computational predictions, GPV was performed to visualize and quantify the experimental flow fields, providing a direct comparison with our CFD results. This technique, previously employed in microfluidic flow studies,^[^ ^37^, ^38^ ^]^ permits the visualization of flow streamlines and quantifying the vortex size. To consistently compare our experimental results for different channel geometries, we fixed the flow conditions by setting the input shear rate was set as *γ_0_* = 1,000 s^−1^. The GPV experiments showed excellent alignment with the CFD simulations, confirming the formation of vortices in the expansion zones (Figure S2A, B, Supporting Information). The velocity vector fields obtained from GPV closely matched the simulated streamlines (Figure S2C,D, Supporting Information), validating our hemodynamic metrics with in vitro assays. The results are particularly effective at capturing the recirculation zones and vortex structures in the β = 120° and 150° geometries.

The double stenosis geometry exhibited a unique flow pattern with two distinct vortices, effectively mimicking more complex geometries found in medical devices.^[^ ^39^ ^]^ This design produced intermediate levels of vorticity compared to the single stenosis designs but created extended regions of disturbed flow that could potentially influence platelet behavior in unique ways. The hemodynamic characterization of our microfluidic devices established a clear link between geometric design and flow patterns (i.e., formation of vortices), providing a solid foundation for interpreting platelet aggregation. This allowed us to attribute observed differences in platelet aggregation behavior to vorticity gradients.

### 2.4 Vorticity‐Related Platelet Aggregation Dynamics

With a thorough understanding of the flow dynamics in our microfluidic devices, we proceeded to investigate how these complex flow patterns influenced platelet aggregation. We perfused whole blood through the devices at an input shear rate *γ*_0_ = 2,000 s^−1^ and monitored platelet aggregation over 300 s using real‐time confocal microscopy. To confirm that the thrombi observed in the expansion zone were formed in situ rather than being transported from upstream locations, we performed whole‐channel imaging experiments (Figure S3 and Video S1, Supporting Information), which revealed minimal platelet aggregation in the upstream region during the 300‐second perfusion period. The resulting platelet aggregates showed distinct patterns across the different β angle geometries (Figure 2B). In all geometries, platelet aggregation was primarily localized to the post‐constriction region. The β = 30° and 60° showed a minimal aggregation in the expansion zone. As the β angle increased, we observed progressively larger aggregates forming in the expansion zone, with β = 150° showing the most extensive aggregation (Figure 2B).

Quantification of thrombus size over time showed that thrombus size increased across all conditions, with the most pronounced growth observed at larger expansion angles, particularly 150°, which showed the highest thrombus size at the 300‐second endpoint (Figure 2C). Smaller expansion angles, such as 30° and 60°, exhibit slower growth and significantly smaller thrombi. The β = 150° produced thrombi were significantly larger by approximately four times than those in the β = 30° at the 300‐second endpoint (Figure 2D). This trend correlated with the maximum vorticity levels observed in our flow characterization studies (Figure 2E; Video S2, Supporting Information), where the vorticity in β = 150° was approximately2,500 times greater than that in β = 30°. Notably, although β = 90° initially exhibited a vorticity level similar to that of β = 30° and 60°, the expansion zone played a pivotal role in the thrombus formation. As aggregates formed, the geometry in this region evolved, eventually creating a separation surface for the streamline. This dynamic transition facilitated the development of a vortex environment akin to that seen in β = 120° and 150° (Figure 2A). In contrast, such transitions were less likely in β = 30° and 60° due to their inherent geometries. Consequently, β = 90° exhibited a platelet aggregation pattern similar to that of β = 120° and 150°, despite its initially lower maximum vorticity level (Figure 2E). These findings suggest there might be a direct relationship between vorticity intensity and platelet aggregation, consistent with relevant observations by other independent groups.^[^ ^27^, ^38^ ^]^

### 2.5 Wall Shear Rate Modulation of Vorticity‐Related Platelet Aggregation

**Figure 3:**
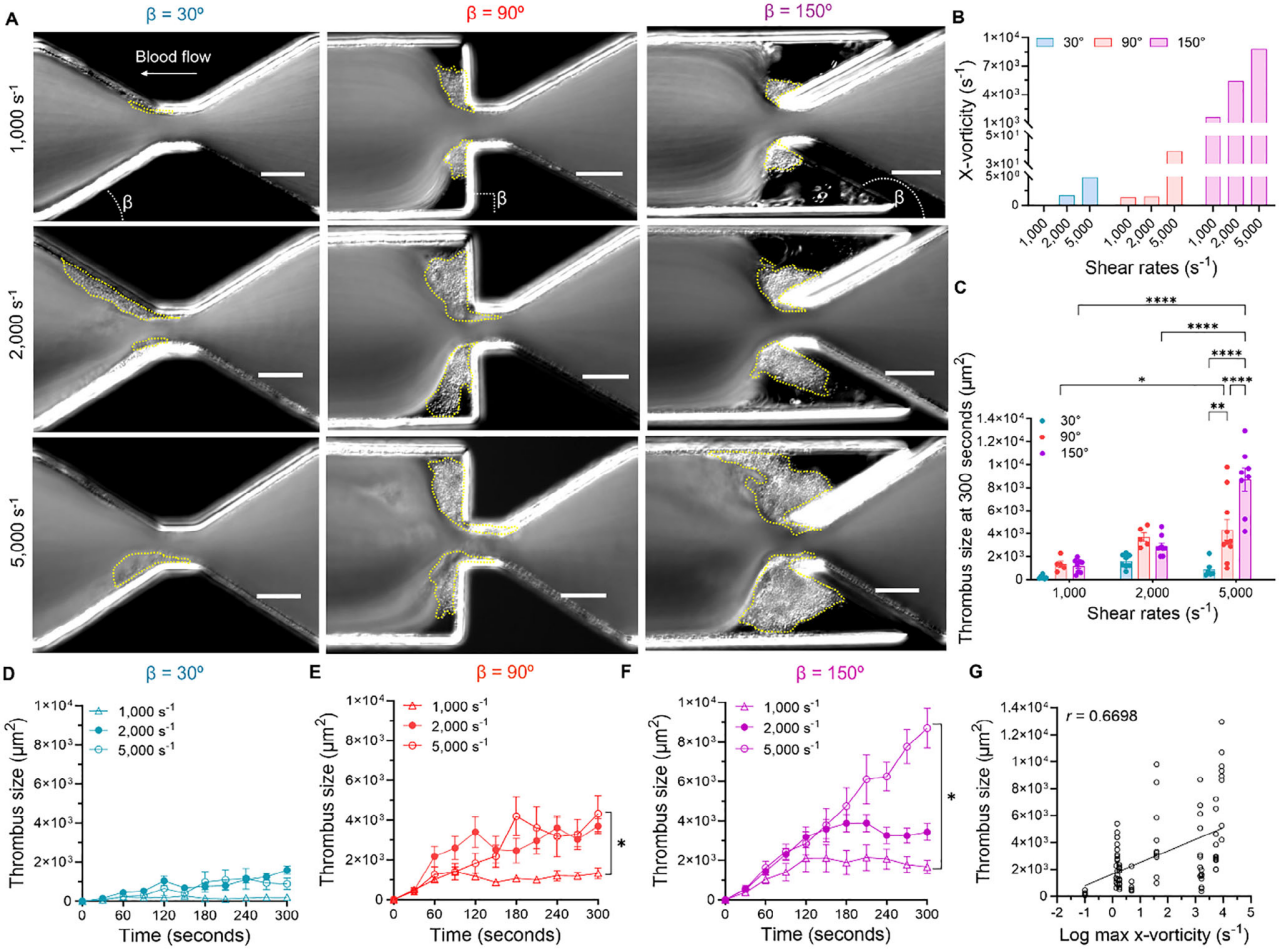
Wall shear rate modulation of vorticity‐facilitated platelet aggregation. A) Representative DIC images and quantification of thrombus size (yellow dotted line) after 300 seconds in β = 30°, 90°, and 150°, subjected to whole blood perfusion at input shear rates of *γ_0_* = 1,000, 2,000, and 5,000 s^−1^. Scale bars: 50 µm. B) Maximum x‐axis vorticity of β = 30°, 90° and 150°, which is above the stenosis apex spanning the shear acceleration and deceleration zones, at the mid‐plane (z‐axis) of the microfluidic. C) The thrombus size of β = 30°, 90°, and 150° at the end point (300 seconds). D–F) Time courses of thrombus growth of β = 30° (D), 90° (E), and 150° (F) under different shear rates. G) Correlation between thrombus size and maximum x‐vorticity. Scatter plot showing the relationship between thrombus size (µm^2^) and the logarithm of maximum x‐vorticity (s^−1^). Each point represents a 300‐second‐endpoint thrombus size from different input shear rate conditions in varying vorticity microfluidic channels, and the solid line indicates the linear regression fit. A positive correlation is observed, with a Pearson correlation coefficient (*𝑟*) of 0.6698, suggesting a strong association between increasing vorticity levels and thrombus size. Data are represented as mean ± s.e.m., n ≥ 3; Statistical analysis: Two‐Way ANOVA with Tukey's post‐test (C), Kruskal‐Wallis with comparisons to *γ_0_* = 1,000 s^−1^ (E‐F) and Simple linear regression (G).^*^ *p* ≤ 0.05, ^**^ *p* ≤ 0.01, ^****^ *p* ≤ 0.0001.

To further elucidate the mechanisms underlying vorticity‐related platelet aggregation, CFD simulations and real‐time blood perfusion with differential interference contrast (DIC) microscopy were performed to investigate the effects of varying shear rates on the process. We tested three input shear rates (*γ*_*0*_ = 1,000, 2,000, and 5,000 s^−1^) for β = 30°, 90°, and 150°, representing the spectrum of our design range (Video S2, Supporting Information). The DIC images at the 300‐second endpoint clearly showed an increase in thrombus size with an increasing shear rate across β = 90°, and 150° (**Figure** **3**A; Video S2, Supporting Information). This effect was most pronounced in β = 150°, where the high shear rate combined with strong vorticity led to extensive platelet aggregation (Figure 3B,C; Figure S4A–D, Supporting Information).

Over the 300‐second thrombus size measurement, thrombus sizes across all conditions (Figure 3C–F) demonstrated that higher shear rates resulted in larger thrombi, with the most pronounced effect observed in β = 150°. Additionally, significantly larger thrombus size and faster aggregation rates were observed at *γ* *_0_* = 5,000 s⁻¹ compared to *γ*_*0*_ = 1,000 s⁻¹ for β = 90° and β = 150°, while no significant difference was noted for β = 30° (Figure 3B,E,F). This suggests a synergistic effect between higher shear rates and stronger vorticity in promoting platelet aggregation. Thrombus formation revealed distinct kinetics for each geometry and shear rate combination (Figure 3D–F): β = 30° showed slow and steady growth with modest size increases at higher shear rates; β = 90° demonstrated rapid initial growth, but ultimately reached a plateau within the observation period; and β = 150° displayed a strong shear rate dependence, with thrombus size increasing significantly as the shear rate rose.

To further investigate the effects of extreme flow conditions on platelet aggregation, we focused on the high input shear rate regime (*γ_0_* = 5,000 s^−1^) across different geometries. The higher shear rates are particularly relevant to certain medical devices, such as mechanical heart valves or ventricular assist devices, where local shear rates can reach these extreme levels.^[^ ^40^ ^]^ CFD simulations at *γ_0_* = 5,000 s^−1^ revealed intense vorticity and shear rate gradients in β = 150° (Figure 3B; Figure S4A, D, Supporting Information). The simulations showed that under these extreme conditions, the vortices became more compact and intense, with higher rotational velocities compared to lower shear rates (Figure S4A, Supporting Information). Notably, β = 150° exhibited rapid and sustained growth throughout the observation period (Figure 3F; Video S3, Supporting Information). The correlation analysis revealed a positive relationship between thrombus size and maximum *x*‐vorticity (*r* = 0.6698), indicating that higher vorticity levels are associated with larger platelet aggregation sizes (Figure 3G). This result highlights the strong influence of vorticity and shear rate on thrombus formation dynamics. Vortex flow, in particular, exhibits an additive effect on amplifying platelet aggregation, especially under higher shear conditions.

### 2.6 Molecular Mechanisms Linked to Vorticity‐Related Biomechanical Platelet Aggregation

**Figure 4:**
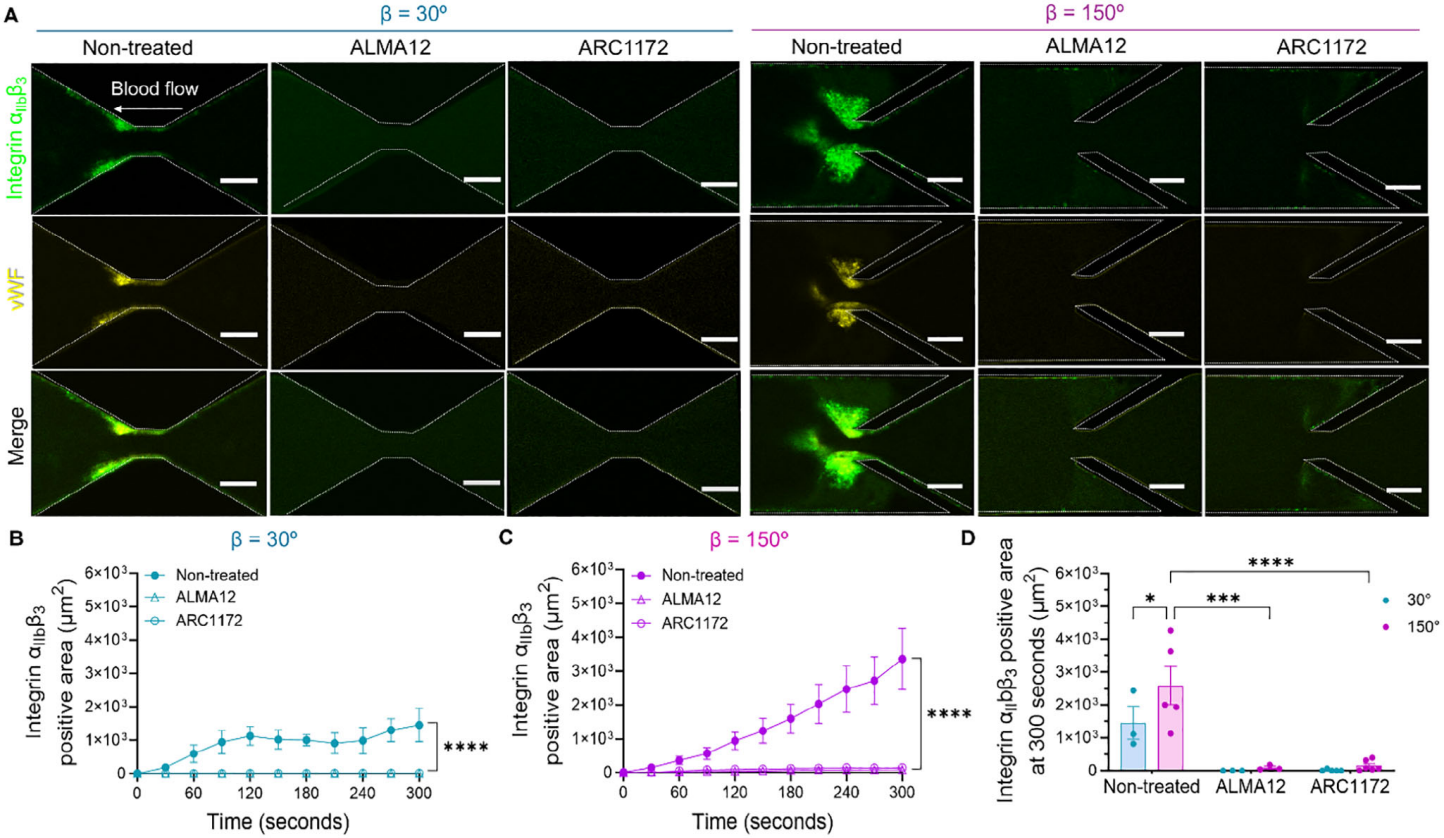
Vorticity‐facilitated in platelet aggregation via vWF–GPIb interaction. A) Representative confocal images of platelet aggregation (anti‐integrin α_IIb_β_3_ mAb P2, *green*) and vWF accumulation (anti‐vWF mAb 2.2.9, *yellow*) in β = 30° and 150° after 300 s of whole blood perfusion. Scale bars: 50 µm. B,C) Time course of P2 positive area (thrombus size) in β = 30° (B) and β = 150° (C). D) Quantification of integrin α_IIb_β_3_ positive area (thrombus size) in β = 30° and 150° after 300 s of whole blood perfusion. All the data are shown at *γ_0_* = 2,000 s^−1^ input shear rate. Whole blood was treated with 25 µg mL^−1^ ALMA12 and 1 µM ARC1172 prior to perfusion. The edge of the channels was marked with white dotted lines. Data are presented as mean ± s.e.m., n ≥ 3; Statistical analysis: Two‐Way ANOVA with Tukey's post‐test (B, C); ^*^ *p* ≤ 0.05, ^***^ *p* ≤ 0.001, ^****^ *p* ≤ 0.0001.

Given the known importance of vWF in high‐shear arterial thrombosis,^[^ ^41^, ^42^ ^]^ we investigated the role of vWF–GPIbα interaction in our vortex‐induced platelet aggregation model. We perfused whole blood in the presence of ALMA12 (anti‐GPIbα blocking antibody) and ARC1172 (anti‐vWF blocking DNA aptamer) at *γ*_0_ = 2,000 s^−1^.^[^ ^43^ ^]^ Confocal microscopy revealed that both ALMA12 and ARC1172 treatment completely abolished integrin α_IIb_β_3_ and vWF levels (**Figure** **4**A,D). Specifically, inhibition of vWF–GPIbα interaction prevents platelet accural from the beginning of the 300‐second microfluidic blood perfusion (integrin α_IIb_β_3_ positive area) in both β = 30° (Figure 4B) and 150° (Figure 4C) configurations. These results demonstrate that the vWF–GPIbα interaction is essential for vorticity mediated biomechanical platelet aggregation.

**Figure 5:**
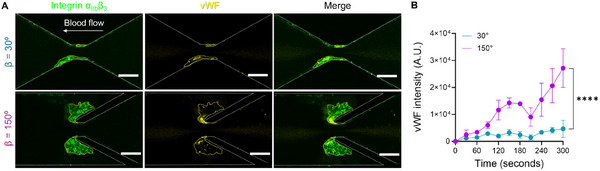
Spatial distribution of vWF accumulation under different vorticity conditions. A) Representative fluorescence images of platelet aggregation (anti‐integrin α_IIb_β_3_ mAb P2, *green*) and spiked recombinant vWF accumulation (rh‐vWF, *yellow*) in β = 30° and 150° after whole blood perfusion at *γ_0_* = 2,000 s⁻¹. Note the distinct vWF localization patterns, with the highest intensities at stenotic edges in both geometries. Scale bars: 50 µm. B) Time course of fluorescence intensity for spike rh‐vWF in β = 30° and β = 150° designs, showing significantly enhanced vWF accumulation in high‐vorticity conditions. All data shown at *γ_0_* = 2,000 s^−1^ input shear rate. The integrin α_IIb_β_3_ positive area is marked with a yellow line. Channel edges are marked with white dotted lines. Data presented as mean ± s.e.m., n ≥ 3; Statistical analysis: Two‐way ANOVA with Tukey's post‐test. ****p ≤ 0.0001.

To further elucidate the spatial distribution and accumulation dynamics of vWF under different vorticity conditions, we employed a “spike‐in” assay using fluorescently‐labeled recombinant human (rh)‐vWF at *γ_0_* = 2,000 s⁻¹. ^[^ ^44^, ^45^ ^]^ Interestingly, vWF accumulation exhibited distinct localization patterns depending on the geometry (**Figure** **5**A). In the β = 30° design, vWF primarily accumulated at the stenotic region, with minimal presence in the downstream expansion zone. In contrast, the β = 150° design showed prominent vWF incorporation throughout the platelet aggregates, but with the highest intensities specifically at the stenotic edges where maximum shear gradients occur. Quantitative analysis of vWF fluorescence intensity over time confirmed significantly enhanced vWF accumulation in the β = 150° geometry (Figure 5B). Notably, despite the formation of large vortices in the β = 150° design, the peak vWF intensity was not within the vorticity area but at the interface of high shear gradients. This spatial distribution suggests that elongational shear components at the stenosis primarily activate and elongate vWF to initiate platelet adhesion, while the rotational shear components in the vortex region subsequently stabilize and promote the growth of platelet aggregates. These distinct but complementary roles of different shear components provide new insights into the complex mechanobiology of vortex‐induced thrombosis.

### 2.7 Platelet Biomechanical Activation Dynamics in Stenotic Vortex

**Figure 6:**
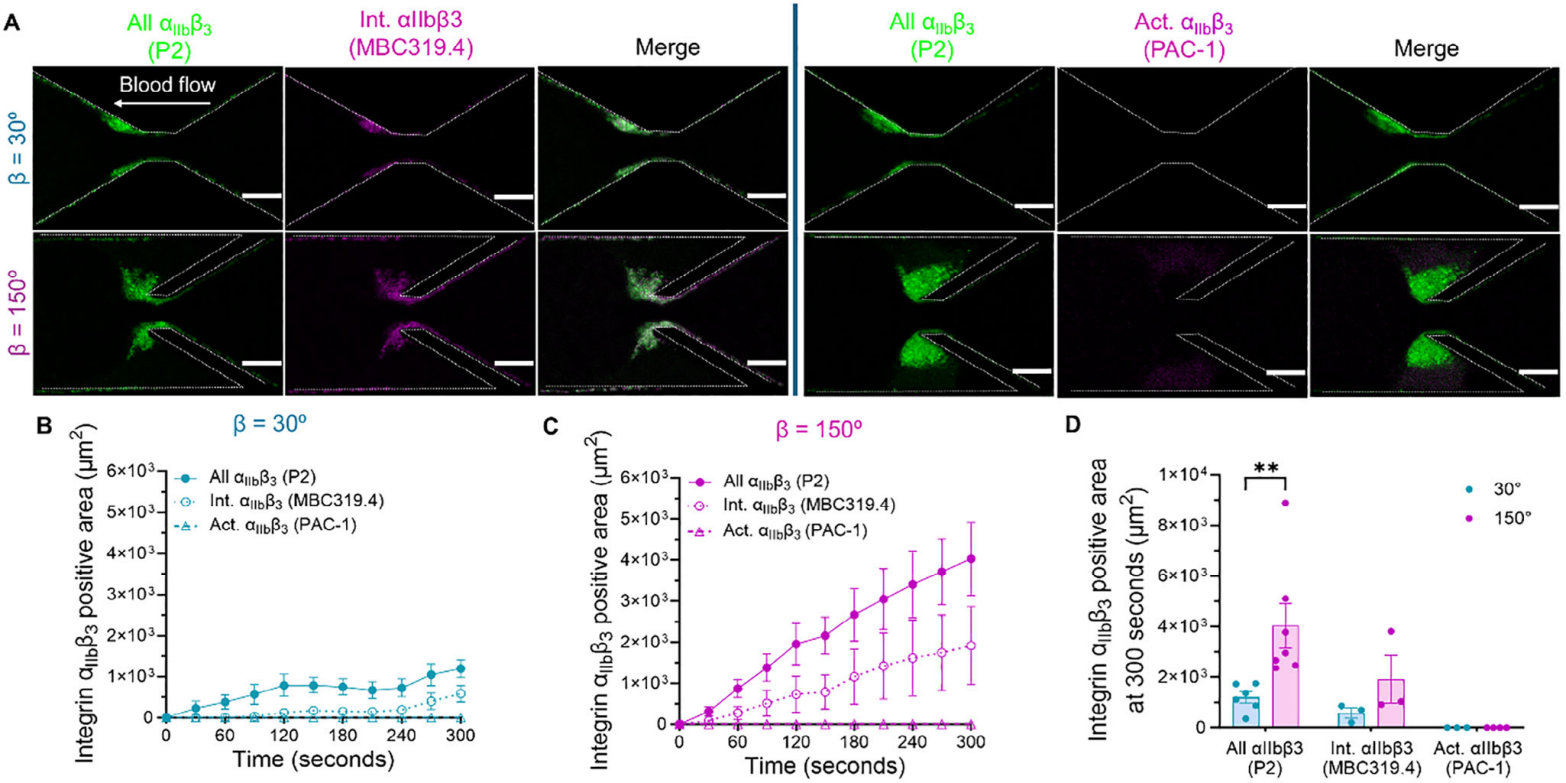
Vorticity‐facilitated in platelet α_IIb_β_3_ integrin intermediate activation. A. Representative confocal images of platelet aggregation (anti‐integrin α_IIb_β_3_ mAb P2, *green*) and activation (MBC319.4 or PAC‐1: *magenta*) at β = 30° and 150° after 300 s of perfusion. Scale bars: 50 µm. B,C. Time course of fluorescence positive areas for all α_IIb_β_3_ (P2), Int. α_IIb_β_3_ (MBC319.4) and Act. α_IIb_β3 (PAC‐1) in β = 30° (B) and β = 150° (C). D. Quantification of fluorescence positive areas for all α_IIb_β_3_ (P2), Int. α_IIb_β_3_ (MBC319.4) and Act. α_IIb_β_3_ (PAC‐1) in the vorticity channels with expansion angles β = 30° and 150° after 300 s of whole blood perfusion. All the data were shown at *γ_0_* = 2000 s^−1^ input shear rate. Channel edges are marked with white dotted lines. Data represents the mean ± SEM, n ≥ 3; Statistical analysis: Two‐Way ANOVA with Tukey's post‐test (D); ^**^ *p* ≤ 0.01.

To further characterize whether vorticity facilitates platelet activation, we examined the activation states of platelets across thrombi. We used the following fluorescently labeled conformation‐specific antibodies against integrin α_IIb_β_3_^[^ ^14^ ^]^: P2 (pan antibody independent of α_IIb_β_3_ conformations), MBC319.4 (intermediate activation antibody, specific to α_IIb_β_3_ extended‐close conformation; Int. α_IIb_β_3_), PAC‐1 (full activation antibody, specific to α_IIb_β_3_ extended‐open conformation; Act. α_IIb_β_3_) (**Figure** **6**A). Time‐lapse immunostaining revealed a rapid increase in both all integrin (P2) and Int. α_IIb_β_3_ (MBC319.4) signals in β = 150°, compared to β = 30° (Figure 6B–D). Interestingly, α_IIb_β_3_ intermediate activation (MBC319.4‐positive area) showed a delayed onset relative to total platelet aggregation, suggesting that initial platelet adhesion precedes integrin activation. This effect was more pronounced in β = 150°, where the MBC319.4‐positive area increased rapidly after an initial lag phase, reaching levels about two times higher than in β = 30° by the 300‐second endpoint. At the 300‐second endpoint (Figure 6A), the total platelet area in β = 150° was around 4 times larger than that in β = 30°, and β = 150° thrombi displayed higher levels of intermediate activation markers. Notably, no Act. α_IIb_β_3_ (PAC‐1) signals were detected in either design within 300 s, suggesting the absence of fully activated α_IIb_β_3_. In both geometries, approximately 40% of platelets displayed intermediate integrin activation, but almost all platelets were PAC‐1 negative (Figure 6B–D).

**Figure 7:**
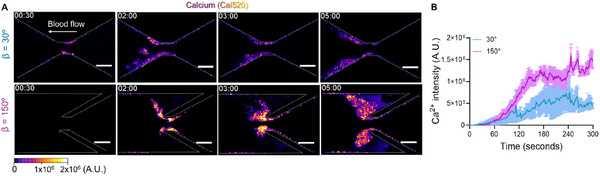
Enhanced platelet calcium mobilization in the stenotic vorticity zone. A) Representative time‐lapse fluorescence images of platelet calcium signaling (*fire LUT*) in β = 30° and β = 150°. Platelets were labeled with Cal520, a calcium‐sensitive fluorescent dye. Scale bars: 50 µm. B) Time‐lapse fluorescence intensity profile of intracellular Ca^2^⁺ intensity over 300 s at 0.33 s intervals to monitor platelet calcium signaling in β = 30° and β = 150°. All the data are shown at *γ_0_* = 2,000 s^−1^ input shear rate. Channel edges are marked with white dotted lines. Data represent mean ± SEM, n ≥ 3; Statistical analysis: Smooth curve.

Calcium triggering plays a critical role in platelet activation and is closely linked to its mechanosensing pathways.^[^ ^47^ ^]^ To further investigate the role of vorticity in platelet activation, we performed intracellular calcium (Ca^2^⁺) imaging using Cal520 — a calcium‐sensitive fluorescent dye. Our results showed that platelets within the vorticity zones in the β = 150° design exhibited a significantly higher rate of intracellular calcium increase. It associated with enhanced platelet aggregation (**Figure** **7**A) during the first 3 min compared to those in the β = 30° design. Additionally, the overall calcium signal was markedly stronger in β = 150°, indicating a more pronounced activation response (Figure 7B). These findings align with previous studies on calcium dynamics in platelets under complex flow conditions.^[^ ^48^ ^]^

Furthermore, to confirm that our observed phenomena are not due to a hypercoagulable state (fibrin formation) induced by blood stasis, we performed additional experiments with anti‐fibrin antibody staining.^[^ ^27^ ^]^ It showed no fibrin signal within the vortex zone during the 300‐second perfusion period (Figure S5, Supporting Information), confirming that the thrombi were formed through biomechanical platelet aggregation driven by vorticity and shear forces, rather than by a hypercoagulable state.^27^

These results suggest that vorticity accelerates the progression of intracellular calcium signaling, enhances platelet aggregation and integrin activation within the forming thrombi, independent of a hypercoagulable state. This indicates that vorticity enhances platelet mechanosensing, likely through more efficient stretching and activation of mechanoreceptor receptors such as GPIbα. However, it does not drive platelets to a fully activated state within 300 seconds (PAC‐1 negative; Figure 5B–D).

## 3 Discussion and Conclusion

Our study presents a microfluidic platform that provides valuable insights into the role of vorticity in platelet aggregation and thrombus formation. By systematically varying the expansion β angle in our double stenosis design, we have successfully characterized the additive effects of vorticity on platelet behavior under physiologically and pathologically relevant flow conditions. Our fabrication technique, employing a double‐casting method combined with critical silanization surface treatment, successfully addressed the challenges of creating high‐expansion‐ratio microfluidic devices with complex geometries, particularly achieving a high aspect ratio at the constriction region. While the double‐casting method has been previously established for the fabrication of high‐aspect‐ratio microstructures,^[^ ^26^, ^28^, ^29^ ^]^ our work demonstrates its successful application and optimization for creating microchannels with extreme expansion angles (up to 150°) that generate controlled vorticity levels. This application of established fabrication techniques to create geometries specifically designed for vorticity studies represents an important contribution to the microfluidic toolbox for thrombosis research. This advancement in microfabrication enables more accurate replication of the intricate flow patterns found in stenotic vessels and medical devices, providing a powerful tool for investigating thrombosis mechanobiology in vitro.

Our findings demonstrate a positive correlation between shear‐induced vorticity and thrombus size (*r* = 0.6698), with higher β angles producing larger and more stable thrombi, especially under extreme shear conditions. Two distinct thrombus formation patterns emerged in our designs: 1) β = 30° and 60°, characterized by the low vorticity but without vortex formation, resulting in slower thrombus growth rates consistently smaller thrombi; 2) β = 90°, 120°, and 150°, which exhibited higher vorticity, including vortex formation, leading to faster thrombus growth rates and significantly larger thrombi. This relationship was particularly evident at high input shear rates, especially at *γ_0_* = 5,000 s^−1^, suggesting a synergistic effect between shear stress and vorticity in promoting platelet aggregation. The observed differences in thrombus formation across the ranges of β angles provide valuable insights into the thrombosis risk associated with various vascular geometries and medical device designs.

The combination of high shear and strong vorticity appears to create ideal conditions for rapid and extensive thrombus formation, mimicking the conditions often found in medical devices associated with thrombotic complications.^[^ ^49^, ^50^ ^]^ The extreme growth of thrombi in the high‐angle designs under these conditions suggests that there may be a threshold effect, where the combination of high shear and strong vorticity triggers a feed‐forward mechanism of platelet activation and recruitment. This phenomenon may have significant implications for the design of medical devices, highlighting the importance of minimizing both high shear regions and strong vortex formation to reduce thrombosis risk.

Our results indicate that the vWF–GPIbα interaction is essential for initiating vortex‐induced platelet aggregation, particularly under high‐shear and high‐vorticity conditions. This is supported by the significant reduction in thrombus formation upon inhibition of this interaction, highlighting the importance of mechanosensing mechanisms in early platelet recruitment.^[^ ^51^ ^]^ The rh‐vWF spike assay further revealed that in high‐vorticity geometries (β = 150°), vWF rapidly accumulates and integrates into platelet aggregates within the vortex region, in contrast to the restricted accumulation in low‐vorticity geometries (β = 30°) — mainly at the stenotic region. These results suggest that vorticity not only enhances vWF–GPIbα binding kinetics but also promotes a broader spatial distribution of vWF during thrombus growth, which could possibly contribute to the thrombus stability in high‐vorticity regions. This enhanced vWF incorporation under vortical flow aligns with the findings from Westin et al. regarding shear‐induced vWF accumulation.^[^ ^52^ ^]^ Further studies are needed to elucidate the mechanisms underlying vWF accumulation in high‐shear or high‐vorticity zones — whether driven by vWF self‐association,^[^ ^53^, ^54^ ^]^ interactions with activated platelets,^[^ ^55^ ^]^ or subsequent engagement with GPIbα^[^ ^10^ ^]^ — and how these processes contribute to thrombus growth and stabilization in such flow environments.

The molecular characterization of platelet activation states using conformation‐specific antibodies to integrin α_IIb_β_3_ provided further insights into the biomechanical nature of vortex‐induced platelet aggregation. The finding that platelets within thrombi predominantly display intermediate activation (MBC319.4‐positive) without progressing to full activation (PAC‐1‐negative) within the 300‐second observation window suggests that vorticity enhances platelet mechanosensing but may not necessarily accelerate the complete activation cascade. Besides, platelet calcium signal experiments revealed that β = 150° exhibited stronger and more sustained calcium signals compared to those in β = 30°. Given the well‐established role of calcium in platelet mechanosensing,^[^ ^47^ ^]^ these findings suggest that vorticity enhances platelet activation by promoting calcium influx. This may be mediated by increased mechanosensory signaling, potentially through GPIbα‐vWF interaction. Moreover, the enhanced calcium response in β = 150° supports the hypothesis that vorticity further amplifies biomechanical platelet aggregation.^[^ ^14^, ^56^ ^]^

Importantly, no fibrin formation was detected in both β = 150° and β = 30° during the 300‐second perfusion, indicating that coagulation was not a contributing factor. This was further confirmed in the control experiments using recalcified citrated blood, where fibrin signals only appeared after 300 seconds, beyond our primary observation window. Together, these data support that thrombus formation in vortex zones is primarily platelet mechanosensing‐driven but not dependent on a hypercoagulable state.

While the vorticity microfluidic platform in this study provides valuable insight into the role of vorticity in platelet aggregation and activation, limitations exist. First, although the simplified microfluidic geometries allow for better experimental control, they are unable to fully reflect the complexities of arterial stenosis or medical devices, which often involve pulsatile and a range of shear rate conditions, and endothelialized or structurally heterogeneous vessel walls. Second, although our results demonstrated a positive correlation between vorticity and thrombus size, the platform cannot distinguish the individual contributions of vorticity, shear gradient, and low shear pocket to thrombus formation, all of which are critical factors in platelet activation and aggregation.^[^ ^3^, ^16^ ^]^ Additionally, longer recording time is required for later stages of thrombus formation to capture the multifactorial nature of thrombus stability. The 300‐second experimental window, which lacks PAC‐1 signal, may not be sufficient for capturing the long‐term dynamic of thrombus growth and the transition from intermediate to full platelet activation.

Our findings have significant implications for understanding thrombosis in medical devices such as ECMO systems, where complex flow patterns and vortices are common at junctions, connectors, and oxygenator components. Clinical evidence indicates that thrombotic events occur in 17–35% of ECMO circuits despite aggressive anticoagulation,^[^ ^2^ ^]^ with thrombosis often initiating at these regions of disturbed flow. Recent work by Goh et al.^[^ ^57^ ^]^ demonstrated that areas of vorticity within ECMO circuits are particularly prone to platelet adhesion and activation, consistent with our observations in the high‐expansion‐angle microfluidic channels. By systematically characterizing the relationship between vorticity and platelet aggregation, our study provides mechanistic insights that could inform the design of more hemocompatible ECMO components with optimized flow geometries to minimize vortex formation.

While our experiments primarily employed constant flow conditions, we recognize that pulsatile flow is a key feature of arterial circulation in vivo.^[^ ^58^ ^]^ Future studies could explore how pulsatility interacts with vorticity to influence platelet aggregation under various pathophysiological conditions. Regarding our chosen shear rate range (1,000 – 5,000 s⁻¹), this is physiologically and pathologically relevant for stenotic regions and the arterial microvasculature,^[^ ^58^ ^]^ although we acknowledge that lower shear rates may be relevant in certain arterial segments, particularly larger vessels and during diastole. Our focus on constant flow conditions is also directly relevant to mechanical circulatory support devices such as ECMO and LVADs, where blood flow is predominantly non‐pulsatile.^[^ ^35^ ^]^

## 4 Experimental Section

### Microfluidic Device Design and Fabrication

Microfluidic channels were designed with a main channel width of 200 µm and height of 70 µm, incorporating an 80% stenosis region followed by an expansion zone. Five different expansion angles (β = 30°, 60°, 90°, 120°, and 150°) were explored, with a fixed contraction angle (α = 30°). A double stenosis design was also included (More details are shown in Supporting Information).

The fabrication process employed an established double casting method previously described: ^[^ ^26^ ^]^

1. Silicon Wafer Preparation: A 6‐inch silicon substrate was cleaned, baked at 150 °C for 20 min, and plasma‐treated to enhance adhesion.
2. Photoresist Application: SU8‐2050 photoresist (MicroChem Crop., USA) was applied using a spin coating at certain (300 rpm, 100 rpm s^−1^, 10 s; 2200 rpm, 300 rpm s^−1^, 30 s) and then soft‐baked at 65 °C for 4 minutes. The pattern was exposed using the MLA100 (HEIDELBERG Instrument), followed by post‐baking at 65 °C for 1 minute and subsequently ramped from 23 °C to 95 °C at 6 °C min^−1^ and held for 6 minutes to ensure controlled heating.
3. Development: The wafer was developed in Propylene Glycol Monomethyl Ether Acetate (PGMEA) for 12 min, cleaned with isopropanol and deionized water IPA, and baked at 120 °C for 3 hours.
4. Double Casting: 1) A negative mold was created, followed by an initial PDMS (3:10 between the curing agent and the base material) casting to create a positive mold. This positive mold underwent silanization surface treatment before the final PDMS casting, following modifications of protocols established by previous studies;^[^ ^28^, ^29^ ^]^ 2) PDMS (Sylgard 184, Dow Corning) was prepared at 1:10 and then poured into the positive mold, degassed, and cured for 2 h at 70 °C in an oven. Inlets (6 mm) and outlets (1 mm) were punched to accommodate flow experiments.

To overcome the challenges posed by the high aspect ratio (4:1 to 5:1) and sharp angles in our design, we utilized this double‐casting approach previously established by Shao et al.^[^ ^29^ ^]^ The efficacy of the silanization process was confirmed through contact angle measurements, which demonstrated a significant change in the wettability of the PDMS surface. After the final casting, the chip was bonded to a glass slide following oxygen plasma treatment (100W for 30 seconds).

### Reagents and Materials

Recombinant human vWF protein (rh‐vWF, His Tag), HPLC‐verified, was obtained from Sino Biological, #10973‐H08C, and reconstituted with PBS to 1 mg mL^−1^. rh‐vWF was labeled with ATTO^TM^ 550 NHS Ester (Sigma Aldrich, #92835‐1MG‐F) to 0.3 mg mL^−1^. Anti‐fibrin antibody (clone: 59D8) was from Gary Matsueda, ^[^ ^69^, ^70^ ^]^ and labeled with Alexa Fluor^TM^ 647 NHS Ester (Thermo Fisher, #A20006‐1MG) 1.5 mg mL^−1^. Cal‐520™ AM was obtained from Abcam, #ab171868, and reconstituted with Dimethyl sulfoxide (DMSO) to 2.5 mM. ALMA12 was from François Lanza (INSERM U.311), 0.9 mg mL^−1^^[^ ^14^, ^71^ ^]^. Aptamer ARC1172 was chemically synthesized (IDT DNA Tech., Coralville, USA) to 1 mM.^43^, ^73^ L

Thrombus profiling reagents were described previously.^[^ ^14^, ^72^ ^]^ CD41 freeze‐dried antibody (P2) was obtained from Beckman Coulter, #IM0145, and reconstituted and labeled with ATTO^TM^ 488 NHS Ester (Sigma Aldrich, #41698‐1MG‐F) to 0.9 mg mL^−1^. Alex Fluor^TM^ 647 anti‐human CD41/CD61 antibody (PAC‐1) was obtained from BioLegend, #362806, 1 mg mL^−1^. Anti‐Integrin β_3_ antibody (MBC319.4) was obtained from Kerafast, #EBW108, and labeled with Alexa Fluor^TM^ 647 NHS Ester (Thermo Fisher, #A20006‐1MG) 0.3 mg mL^−1^. Anti‐vWF 2.2.9 antibody was obtained from MERU, and labeled with ATTO^TM^ 550 NHS Ester (Sigma Aldrich, #92835‐1MG‐F) to 1 mg mL^−1^.

Clexane was obtained from Sanofi Medical, 100 mg mL^−1^ (10 000 U). Anticoagulant 3.8% sodium citrate was house made with sodium citrate dihydrate.^14^, ^73^ After collecting blood from donors, allow blood to rest for at least 15 min at 37 °C and then incubate with different inhibitors for 30 min at 37 °C before perfusion.^43^

### Blood Collection and Treatment

Blood collection from healthy donors was approved by the University of Sydney Human Research Ethics Committee (HREC, project 2023/HE000582). Donors provided written informed consent and were screened for appropriate age, weight, and absence of anticoagulant or anti‐inflammatory medication use 72 h prior to donation. Blood was collected via a 19G butterfly needle, slowly drawn into syringes containing anticoagulants of 20 U mL^−1^ Clexane and 3.8% w v^−1^ sodium citrate, respectively. The blood was then rested in a 37 °C dry incubator for at least 15 min before using. 3.8% w v^−1^ sodium citrate whole blood was recalcified with 6 mM CaCl_2_ right before perfusion. For most experiments, whole blood with retained red blood cells (RBCs) was used directly for perfusion experiments within 4 h of collection.

For experiments requiring platelet‐rich plasma (PRP), Clexane‐treated whole blood was supplemented with 0.02 U mL^−1^ apyrase and allowed to rest for 30 min before centrifugation. The blood was then centrifuged at 200 g for 10 min at 37 °C. PRP was carefully collected while avoiding the buffy coat, which was subsequently collected and then discarded, leaving the RBC pellet. Both PRP and RBCs were allowed to rest for 15 min. Next, Cal520 (2.5 µM) was added to PRP and incubated at 37 °C for 30 min. Finally, PRP was reconstituted with RBCs to achieve a final hematocrit of 40% and a platelet concentration of around 2 × 10⁸ mL^−1^ for perfusion.

For immunostaining staining in microfluidic perfusion experiments, whole blood was pre‐incubated for 15 min at 37 °C with anti‐α_IIb_β_3_ antibodies—P2 (1 µg mL^−1^), PAC‐1 (10 µg mL^−1^), MBC319.4 (2 µg mL^−1^), anti‐vWF mAb 2.2.9 (7 µg mL^−1^) or anti‐Fibrin (0.5 µg mL^−1^). Given that the anti‐vWF 2.2.9 antibody used targets the C‐terminal region (residues 1366 – 2050) of mature vWF,^[^ ^59^ ^]^ which overlaps with or was adjacent to the A1 and A2 domains—critical for shear‐dependent unfolding and GPIbα binding,^[^ ^10^ ^]^—antibody accessibility may be limited, particularly in low shear regions such as the vortex. This limitation could impair the detection of vWF–platelet binding events. To quantify vWF accumulation in the stenotic region accurately, mAb 2.2.9 was replaced with ATTO^TM^ 550‐labelled rh‐vWF (30 µg mL^−1^) before perfusion as a vWF “spike‐in” assay. For antithrombotic drug testing, blood was pre‐incubated with the ALMA12 (25 µg mL^−1^) and ARC1172 (1 µM) for 30 min at 37 °C.

### Real‐Time Thrombosis Visualization and Quantification

Blood perfusion experiments were conducted using PDMS chips coated with rh‐vWF (50 µg mL^−1^) for 1 h at 37 °C. Whole blood samples were perfused using a Legato^®^ 111 syringe pump (KD Scientific, USA) at flow rates corresponding to input shear rates of *γ_0_* = 1,000, 2,000, or 5,000 s^−1^, with experiments lasting 300 seconds. Platelet aggregation was monitored in real‐time using differential interference contrast (DIC, Olympus IX83, Australia) and confocal microscopy (Olympus FV3000RSAustralia), and quantified by measuring aggregate or fluorescence coverage area, which localized at the PDMC wall, using ImageJ. The real‐time and end‐point areas of platelet aggregation were determined for the channel wall within the observation area. Aggregation area analysis was corrected based on confocal imaging fields.

To confirm that the thrombi observed in the expansion zone were formed in situ rather than being transported from upstream locations, additional whole‐channel imaging experiments were performed using a low magnification objective (Olympus IX83Australia). These experiments enabled visualization of around 1.5 mm of the microfluidic channel, allowing us to track platelet behavior from the inlet region through the stenosis and into the expansion zone.

Thrombus composition and platelet activation were assessed via rh‐vWF “spike‐in” assay and fluorescence staining for platelet activation markers, including P2 (all integrin α_IIb_β_3_), MBC319.4 (intermediate α_IIb_β_3_ activation), PAC‐1 (fully activated α_IIb_β_3_), and 59D8 (anti‐fibrin). Real‐time imaging was performed using an Olympus IX83 inverted microscope (20×, *NA* = 0.70) for DIC and an Olympus FV3000RS confocal microscope (40×, *NA* = 0.95), with the focal plane set 30 µm above the coverslip. Platelet and vWF signals were acquired with a default pinhole size and 15‐frame averaging. Quantitative analysis of fluorescence signals was conducted frame‐by‐frame using ImageJ.

### Computational Fluid Dynamics (CFD) Simulations

To quantify and map out the hemodynamic distributions and how vortical structures influence thrombus formation, CFD simulation was performed utilizing ANSYS Fluent 2023 R3 (version 20.1; Canonsburg, PA, USA). The microfluidic geometries were designed in ANSYS Spaceclaim with inlet and outlet dimensions of *X*_*0*_ = 1,000 µm length, *Y*_*0*_ = 200 µm width, and *Z*_*0*_ = 70 µm height, with a constriction zone of 40 µm in width, produced by varying angles (Figure S1A, Supporting Information). Smooth transition inflation layers were applied along the entire length of the channel using a growth rate of 1.2 with a maximum of 10 layers, generating > 500 000 elements deployed along the flow channel with an element quality > 0.99.

The flow was assumed to be steady and laminar, and the fluid was considered Newtonian with constant properties, which was appropriate for the high shear rates (*γ_0_* >1,000 s^−1^) used in this study as established by previous research.^[^ ^60^ ^]^ While it is acknowledged that blood contains various cells and particles that can influence local flow dynamics, at these high shear rates, the differences between Newtonian and non‐Newtonian blood flow models become minimal.^[^ ^60^, ^61^, ^62^ ^]^ The flow rate was selected to be 9.8, 19.6, and 49 µL min^−1^ to achieve bulk input shear rates of *γ_0_* = 1,000, 2,000, and 5,000 s^−1^, respectively. In ANSYS FLUENT, the standard second‐order scheme as well as the second‐order upwind scheme were applied to discretize the pressure and momentum equations, with the coupled algorithm utilized for pressure‐velocity coupling. The simulation was considered converged when the residuals reached 1e^−6^. In CFD‐POST, shear rate and vorticity contours were plotted from the centre plane of the geometry to reflect experimental conditions. Flow streamlines were also plotted, showing the vortical structures produced in each microfluidic channel. The streamline depicting the maximum vortex deviation was isolated, and the position, *x*‐vorticity, and shear rate gradient metrics were plotted in a line graph.

### Ghost Particle Velocimetry (GPV)

GPV, an optical method leveraging speckle patterns generated by high‐refractive‐index nanoparticles,^[^ ^63^, ^64^ ^]^ was employed to assess flow behavior in stenosis geometries with β angles of 30°, 60°, 90°, 120°, and 150°. GPV experiments were performed to validateCFD simulations of flow characteristics across various β angles within microfluidic channels. (More details are shown in Supporting Information)

To enable vortex detection and flow pattern analysis, a 0.2% v v^−1^ suspension of 200 nm polystyrene nanoparticles (Sigma‐Aldrich, USA) was prepared as tracer particles. This suspension was perfused into the microfluidic channels using a syringe pump (Legato 270, KD Scientific, USA) at flow rates producing a bulk input shear rate of *γ_0_* = 1,000 s⁻¹, ensuring compatibility with CFD simulation conditions.

Bright‐field microscopy through an inverted optical microscope (Nikon Eclipse Ti2‐U, Japan) was used to capture the speckle patterns generated by the nanoparticles. This originates from a thin fluid layer within the microfluidic channel whose thickness can be controlled by the numerical aperture of the condenser lens.^[^ ^38^ ^]^ All our experiments extract GPV data from the middle plane of the microchannel.

Image sequences were collected using a high‐speed camera (Photron Nova S12, Japan) at different frame rates, up to 50,000 frames per second (fps), depending on the local flow velocity. The images were processed using ImageJ to enhance the speckle pattern by subtracting the median of several hundred images from each frame to remove any static contribution.^[^ ^37^ ^]^ Finally, a cross‐correlation analysis was performed using PIVlab,^[^ ^65^ ^]^ an open‐source MATLAB routine, to extract the velocity profiles, the streamlines, and to quantify the vorticity.

The speckle pattern images were processed using PIVlab (Thielicke and Stamhuis, 2014), which implements a cross‐correlation algorithm similar to those used in traditional PIV. The cross‐correlation between corresponding windows in consecutive frames was calculated to determine the local displacement vector. The resulting vector fields were filtered using standard validation criteria (median filter with threshold of 2 pixels) to remove spurious vectors. The final velocity fields were calculated by dividing the displacement vectors by the time interval between frames.

To compare experimental results with CFD simulations, the GPV‐derived velocity fields were normalized by the maximum velocity and overlaid with the CFD‐predicted streamlines. A quantitative comparison was performed by extracting velocity profiles along selected transects in both the experimental and simulated data.

### Statistical Analysis

All experiments were performed in triplicate unless otherwise stated. Data were presented as mean ± standard error of the mean (s.e.m.) of n ^3^ 3. Statistical significance was determined using the Kruskal‐Wallis test to compare multiple independent groups, or one‐way ANOVA or two‐way ANOVA followed by Tukey's post‐hoc test for multiple comparisons, or Simple linear regression for correlation analysis. Fluorescent signals of spiked rh‐vWF and intracellular calcium within platelet aggregates were analyzed after background subtraction; The calcium mobilization curves were smoothed to assess calcium influx dynamics of platelet aggregates. P‐values ≤ 0.05 were considered statistically significant. All statistical analyses were performed using GraphPad Prism 10 software.

## Conflict of Interest

The authors declare no conflict of interest.

## Author Contributions

J.R. performed blood perfusion, cast PDMS chips, conducted analysis and interpretation of data, and organized the figures. N.A.Z.A. performed microfluidic blood perfusion, immunostaining, and data analysis. A.D. performed data analysis for immunostaining. A.S. and Y.C.Z. performed the CFD simulation and data analysis, and interpretation. A.S., R.G., and A.N. designed the chips, developed the soft lithography method, refined the PDMS casting method, and produced microfluidic devices. Y.C. performed the GPV simulation and data analysis. Z.W. developed the microfluidic pump to produce pulsatile flow. M.C.L.W., Q.P.S., D.V., and A.W. provided critical suggestions, comments, and corrections to the manuscript. M.C.L.W. assisted in data interpretation and figure visualization and co‐supervised the study. L.A.J. is the senior author, proposed research ideas, designed the experiments, and supervised the study. All authors contributed to the manuscript writing.

## Supporting information



Supporting Information

Supplemental Video 1

Supplemental Video 2

Supplemental Video 3

Supplemental Video 4

## Data Availability

The data that support the findings of this study are available from the corresponding author upon reasonable request.
