# Supplementary material for: Vorticity‐Facilitated Platelet Aggregation: a High Expansion‐Ratio Stenotic Microfluidic Platform Unravels the Role of Complex Flow Dynamics in Arterial Thrombosis
Source: Adv Healthc Mater. 2025 Jun 4;14(28):2500436. doi: 10.1002/adhm.202500436 (PMC12581891; doi:10.1002/adhm.202500436)
Supplement: Supplementary file 1 — Supporting Information [file ADHM-14-0-s004.docx]

**Supporting Information**

Title: Vorticity-facilitated platelet aggregation: A high expansion-ratio stenotic microfluidic platform unravels the role of complex flow dynamics in arterial thrombosis

Jianfang Ren, Nurul Aisha Zainal Abidin, Allan Sun, Rui Gao, Yuxin Chen, Arian Nasser, Zihao Wang, Yunduo Charles Zhao, Alexander Depuy, Anna Waterhouse, Qian Peter Su, Daniele Vigolo, Mike Chia Lun Wu, Lining Arnold Ju*

**Fabrication methods**

The fabrication process for the Vorticity series microchannels has been divided into two groups. The first group (β = 30°, 60°, 90° and 120°) was fabricated using PDMS casting from a patterned mold created through standard photolithography techniques. The second group (β = 150°) was fabricated using a double casting method to achieve sharp angle with high expansion microstructures. All photolithography was conducted using a maskless aligner (MLA100, Heidelberg Instruments).

For the first group (β = 30°, 60°, 90° and 120°), a 6-inch single polished silicon wafer was spin coated with SU8-2050 photoresist (MicroChem Corp.) using a spread cycle of 300 rpm and 100 rpm s^-1^ for 10 s and a development cycle of 2,200 rpm and 300 rpm s^-1^ for 30 s. The SU8-2050-coated wafer was soft-baked at 65 °C for 4 minutes, with the temperature subsequently ramped at a rate of 6 °C min^-1^ to 95 °C, where it was held for 6 minutes to ensure complete solvent evaporation. After removing the wafer from the hotplate, it was allowed to cool to room temperature. The patterned area of the SU8 film was exposed using the MLA100 system with UV light at a wavelength of 365 nm, selectively exposing the non-channel regions.. Following exposure, the patterns were cross-linked by post-exposure baking on a hotplate to stabilize and enhance the polymer cross-linking. This starts with 1 minute at 65 °C. The temperature was subsequently ramped at 6 °C min^-1^ to 95 °C, where it was maintained for 6 minutes. This ramping process, initiated from 23°C, ensured controlled thermal conditions. The exposed and cross-linked film gradually cooled to room temperature on the hotplate to minimize thermal stress and prevent potential cracking from abrupt temperature changes. The unexposed SU8 was developed in Propylene Glycol Monomethyl Ether Acetate (PGMEA) for 12 minutes with periodic agitation to effectively remove the unexposed material, resulting in designated channels in the group 1. After developing the SU8 pattern, the wafer was thoroughly rinsed with isopropanol and deionized water. To enhance the mechanical stability and durability of the cross-linked SU8 pattern, a final hard bake was performed at 120 °C for 3 hours. The resulting SU8 pattern was subsequently used as a mold, onto which PDMS was cast following standard fabrication procedures.

For the second group (β = 150°), the high expansion ratio of the structures and the sharp, elongated design in the α = 30°, β = 150° chip pose significant challenges during fabrication. Specifically, the sharp corners of the embedded structure complicate the pattern formation process, often leading to incomplete or defective features. The high aspect ratio of the sharp-cornered structure frequently results in fabrication failures during the development process, even with optimized photolithography parameters as optimized above. If the exposure dose is insufficient, the SU8 photoresist does not adequately bond, causing the entire structure to detach during development. Conversely, excessive exposure dose results in incomplete development in the sharp and narrow regions, leading to similar fabrication failures.

To address this challenge of the sharp angle fabrication, a double-casting method was applied and implemented to meet the unique design specifications. In this process, the positive mold is first converted into a negative mold to accurately reproduce the sharp corners of the embedded structures, as illustrated in Figure 1B. This is achieved by switching the UV expose regions to the previously non-exposed channel region exclusively for the group 2 (β = 150°). Compared to eliminating the photoresist in long, narrow areas to form the structure (positive mold), retaining the photoresist in these regions while removing it from other areas significantly simplifies the fabrication process (negative mold). However, this approach results in an SU8 mold that cannot be directly used for microfluidic chip preparation through conventional mold casting. Instead, the initial mold must serve as an intermediate to produce a secondary mold, which is then used to achieve the desired structure.

This modification significantly enhanced the development process compared to using a positive mold. Following the lithography process, the positive mold was fabricated through an initial PDMS (mix ratio of curing agent and PDMS is 3:10) casting. To generate PDMS channel, a silanization surface treatment was applied to prevent adhesion between the PDMS structures and the PDMS positive mold. This treatment ensured the formation of a uniform separation layer on the mold surface. To verify the successful incorporation of the separation layer, the contact angle of the treated PDMS mold surface was measured using a drop shape analyzer (Theta Flex Auto 2). The contact angle measurements confirmed that the silanization process effectively altered the surface wettability of the PDMS mold (Figure S2B, Supporting Information 2). After the surface treatment, the second PDMS casting could be easily separated from the mold. The double-casting process is illustrated in Figure. 1B.

Once the mold was prepared, PDMS and its curing agent were mixed at a 10:1 ratio and degassed for 30 minutes to remove air bubbles. The degassed mixture was then poured onto the prefabricated PDMS mold and cured in an oven at 80°C for 20 minutes. After curing, the PDMS channels were carefully peeled off the SU8 mold. A 6 mm inlet reservoir hole was created using a biopsy punch, while a 2 mm biopsy punch was used for the outlet connection to the syringe pump. Once both holes were prepared, the PDMS channel was placed onto a 65 × 22 mm glass slide. The adhesion between the PDMS and glass was achieved due to the inherently low surface energy of PDMS.

**Technical discussion**

Using standard photolithography techniques combined with double-casting technology combined with the established double-casting approach, a series of vorticity microchannel chips were successfully fabricated. SEM images of the fabricated microchannels are presented in Figure S1B (Supporting Information 2). The fabrication of sharp angles with high-expansion ratio structures typically requires careful optimization of photolithography parameters, including exposure dose, defocus, soft-bake temperature, and post-bake temperature. Despite extensive optimization, the fabrication of the structure of β = 150° initially proved unsuccessful. By employing the double-casting method, which involves reversing the lithography pattern, the fabrication process demonstrated significantly improved robustness, ultimately enabling the successful production of the 30° structures. Compared to traditional high-expansion-ratio manufacturing techniques, which demand strict parameter optimization, the double-casting method reduces the complexity of the development process by eliminating the need for such rigid constraints.

**Analysis of Flow Profile and Vorticity Using Ghost Particle Velocimetry (GPV)**

Ghost Particle Velocimetry (GPV) is a recently developed velocimetry technique designed to measure fluid flow velocities in small-scale devices by utilizing the speckle pattern generated by nanoparticles. This method employs bright field optical microscopy, offering a cost-effective and accessible method to study complex fluid dynamics while maintaining a rigorous approach. GPV is particularly valuable for micro/milli-fluidic systems applications, such as analyzing vortical structures in stenotic junctions as in the present work.^[1,2]^

The experimental configuration for GPV is centered around a Nikon inverted optical microscope (Eclipse Ti2-U, Japan) provided with a white LED light source. To produce the speckle pattern essential for GPV, the numerical aperture of the condenser lens (*NA*_c_) is adjusted by manually closing the condenser aperture diaphragm to a value of approximately 0.15-0.20, transforming the incoherent light into a partially coherent source within a thin volume of the sample that enables speckle formation. The thickness, $\delta$, of this volume is linked to the NA_c_ by the relationship $\delta=\lambda/NA_{c}$, in which $\lambda$ is the illumination source wavelength.

To visualize the flow pattern, the tracers used in GPV are 200 nm polystyrene particles (Sigma-Aldrich), selected for their size, which falls below the diffraction limit (~300 nm), and their relatively high refractive index (~1.55). This ensures that the particles are invisible in raw images but capable of producing a detectable speckle pattern under appropriate illumination conditions. The particles are suspended in deionized water at a concentration of 0.2% w/w. This low concentration prevents particle interactions or flow disturbances, as verified by a Stokes number ranging from 10⁻³ to 10⁻⁴, well below unity, indicating negligible inertial effects.

A high-speed camera (Photron Nova S12, Japan) is directly mounted onto the microscope to capture the dynamic speckle patterns generated by the nanoparticles within the flow, ensuring high temporal resolution of the fluid motion. The frame rate is adjusted to the flow velocity so that the cross-correlation analyses can be performed accordingly (see below for more details). The fluid, seeded with nanoparticles, is introduced into the microfluidic device via a Legato 270 syringe pump (Legato 270, KD Scientific), which maintains a constant volumetric flow rate. For the devices with different expansion angles, the inlet flow rates, corresponding to a shear rate γ_0_ of 1,000 s^-1^, were used to pinpoint the significant fluid-dynamic features. The fluid delivery system is completed with 1.6 mm inner diameter Tygon Microbore tubing (COLE-PARMER), connecting the syringe to the device and collecting the exiting fluid.

To ensure data quality, the particle suspension is filtered using a 0.45 μm syringe filter (Sigma-Aldrich) prior to each experiment to reduce impurities. Any residual impurities appearing in the images are addressed during analysis, as their velocity vectors typically deviate significantly from those of the speckle pattern and can be excluded from the final results.

The GPV experimental process involves the following key steps:

1. Image Acquisition: The high-speed camera records a continuous sequence of frames at a fixed frame rate (FR), capturing the speckle pattern created by the nanoparticles. The time interval between consecutive frames is then calculated as the inverse of the frame rate (1/FR).
2. Image Preprocessing: In the raw images, the speckle pattern is obscured by the dominance of the transmitted light. To isolate the speckle pattern, the median of a large set of frames (up to 300 frames in this study) is computed and subtracted from each image in the sequence using ImageJ.^[3]^ This step eliminates the static background, revealing the dynamic speckle pattern.
3. Velocity Field Reconstruction: The preprocessed image sequence is analyzed using cross-correlation techniques to reconstruct the two-dimensional (2D) flow velocity field. Frame pairs (e.g., 1-2, 2-3, 3-4, etc.) are generated from the continuous sequence to serve as the basis for the cross-correlation analysis.

The velocity field analysis in GPV is conducted using PIVlab,^[4]^ an open-source MATLAB® routine tailored for velocimetry studies. The cross-correlation process is configured to use a double-pass correlation with the first pass interrogation window set to 32 × 32 pixels, and the second pass set to a reduced interrogation window of 16 × 16 pixels. We used the Fast Fourier Transform (FFT) window deformation cross-correlation with linear window deformation algorithm and set the correlation step size to half the interrogation window size, enhancing spatial resolution.

These settings optimized the balance between accuracy and resolution, enabling the detailed capture of the flow features. The output from PIVlab is a .csv file containing the planar velocity components at each point in the imaged plane. Custom MATLAB® scripts are then employed to process these data files and generate editable contour plots of the velocity fields for visualization and interpretation.

**References**

[1] Z. Schofield, H. A. Baksamawi, J. Campos, A. Alexiadis, G. B. Nash, A. Brill, D. Vigolo, *Commun Mater* **2020**, *1*, DOI 10.1038/S43246-020-00066-2.

[2] M. Riccomi, F. Alberini, E. Brunazzi, D. Vigolo, *Chemical Engineering Research and Design* **2018**, *133*, 183.

[3] C. A. Schneider, W. S. Rasband, K. W. Eliceiri, *Nature Methods 2012 9:7* **2012**, *9*, 671.

[4] W. Thielicke, E. J. Stamhuis, *J Open Res Softw* **2014**, *2*, DOI 10.5334/JORS.BL.

**Supplementary Figures**


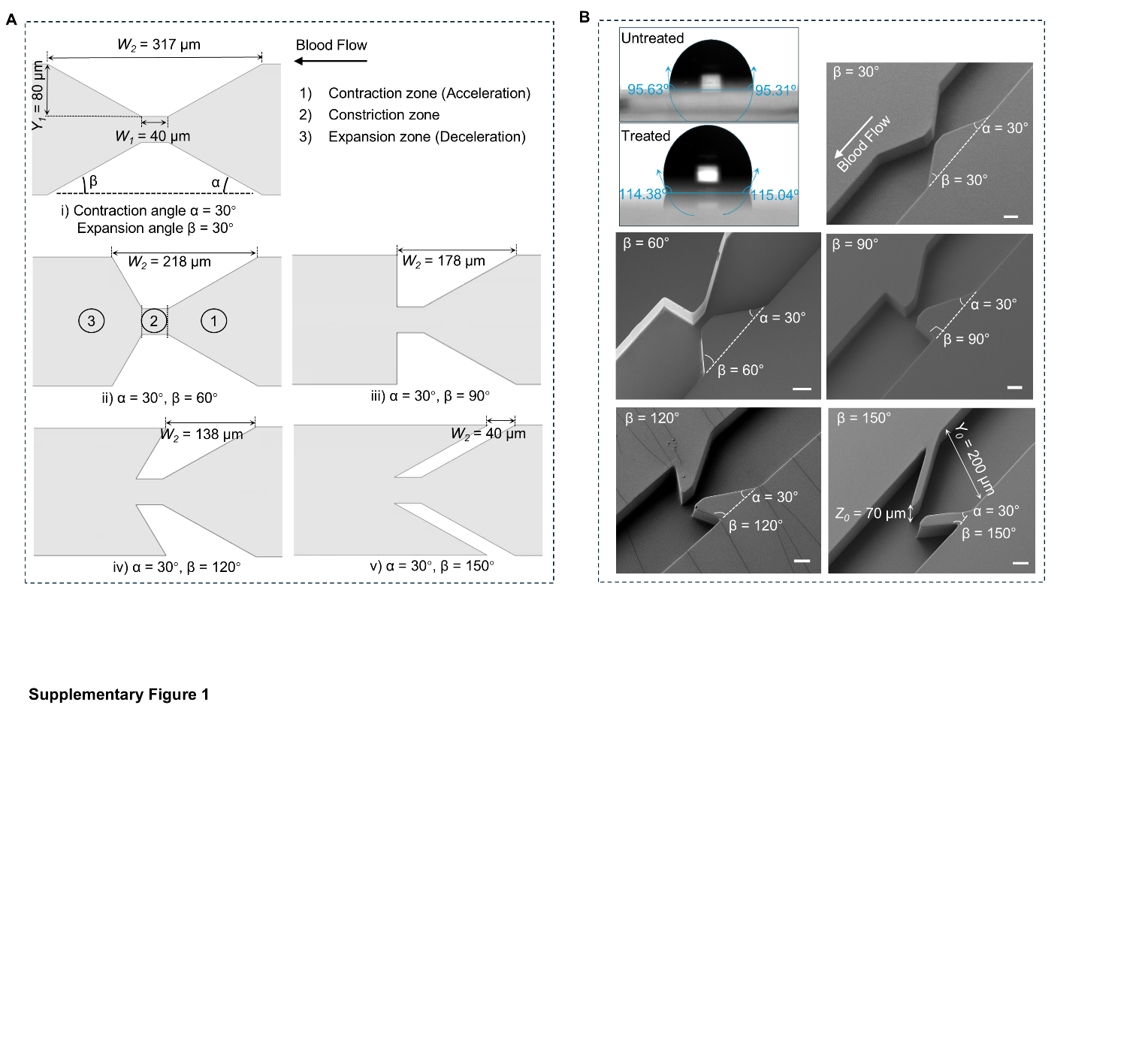


**Supplementary Figure S1. SEM images of the vorticity microfluidic devices. A**) Schematic representation of microchannel geometries with varying expansion angles (β) and fixed contraction angle (α = 30º). The microchannel design consists of three distinct regions: (1) the contraction zone, where blood accelerates as it enters the constriction; (2) the constriction zone, characterized by the narrowest width of 𝑊*_1_*=40 𝜇𝑚; and (3) the expansion zone, where flow decelerates as the channel widens. (i–v) Illustrations of the microchannel geometries with expansion angles of β = 30º, 60º, 90º, 120º, and 150º, resulting in expansion widths (𝑊*_2_*) of 317 µm, 218 µm, 178 µm, 138 µm, and 40 µm, respectively. B) The difference in the contact angle of the silanization-treated PDMS surface compared to the untreated surface, Trichloro(1H,1H,2H,2H-perfluorooctyl) silane layer increases the contact angle, which indicates the deposition layer is loaded successfully; and SEM images of microfluidic channels, with 80% reduction of the width of *Y_0_*  = 200 µm and the height of *Z_0_  =* 70 µm. Scale bars: 50 µm.


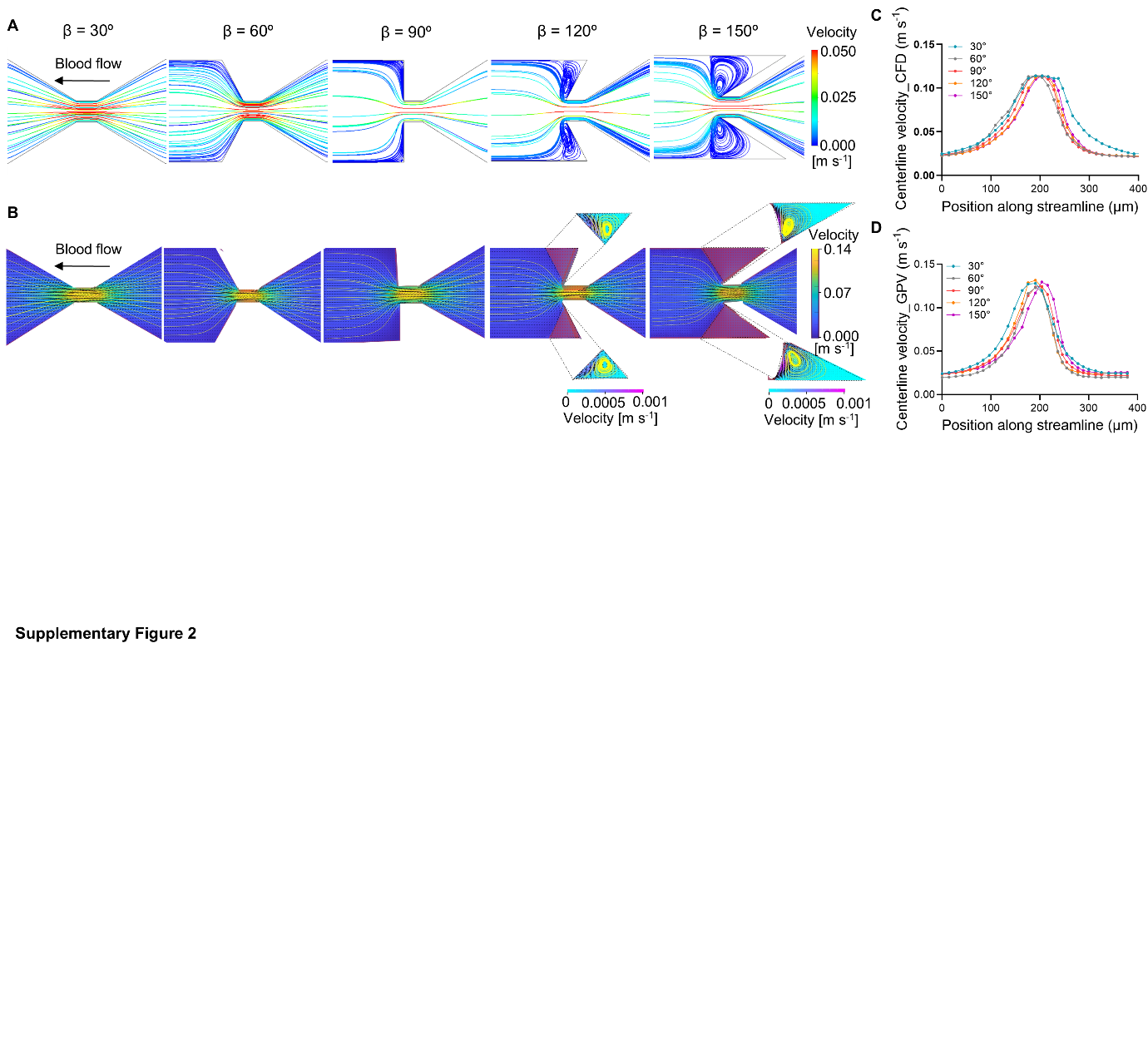


**Supplementary Figure S2. Hemodynamic characterization with Computational Fluid Dynamics (CFD) and correlation with Ghost Particle Velocimetry (GPV) for vorticities.**

All experiments were done under input shear rate *γ*_0_ = 1,000 s^-1^. A) CFD simulated velocity streamlines at the mid-plane (*z*-axis) of the microfluidic channels. B) Velocity vector field obtained using GPV at the mid-plane (*z*-axis) of the channels, tracking the speckle patterns formed by the interference of the bright field light with the nanoparticle tracers. Bright-field image frames were processed by subtracting the median image of over 100 frames to generate a speckle pattern. Streamlines are shown in yellow. The main flow regions represented by a blue-to-yellow velocity gradient (0 - 0.14 m s^-1^) where vortex regions (insets) post-stenosis at larger expansion angles (β = 120°, 150°) represented by aqua-to-purple gradient (0 – 0.001 m s^-1^). The vortex regions match with CFD predictions. C-D) Line graphs of the predicted streamline velocity distributions of different channels from CFD (C) and GPV (D).


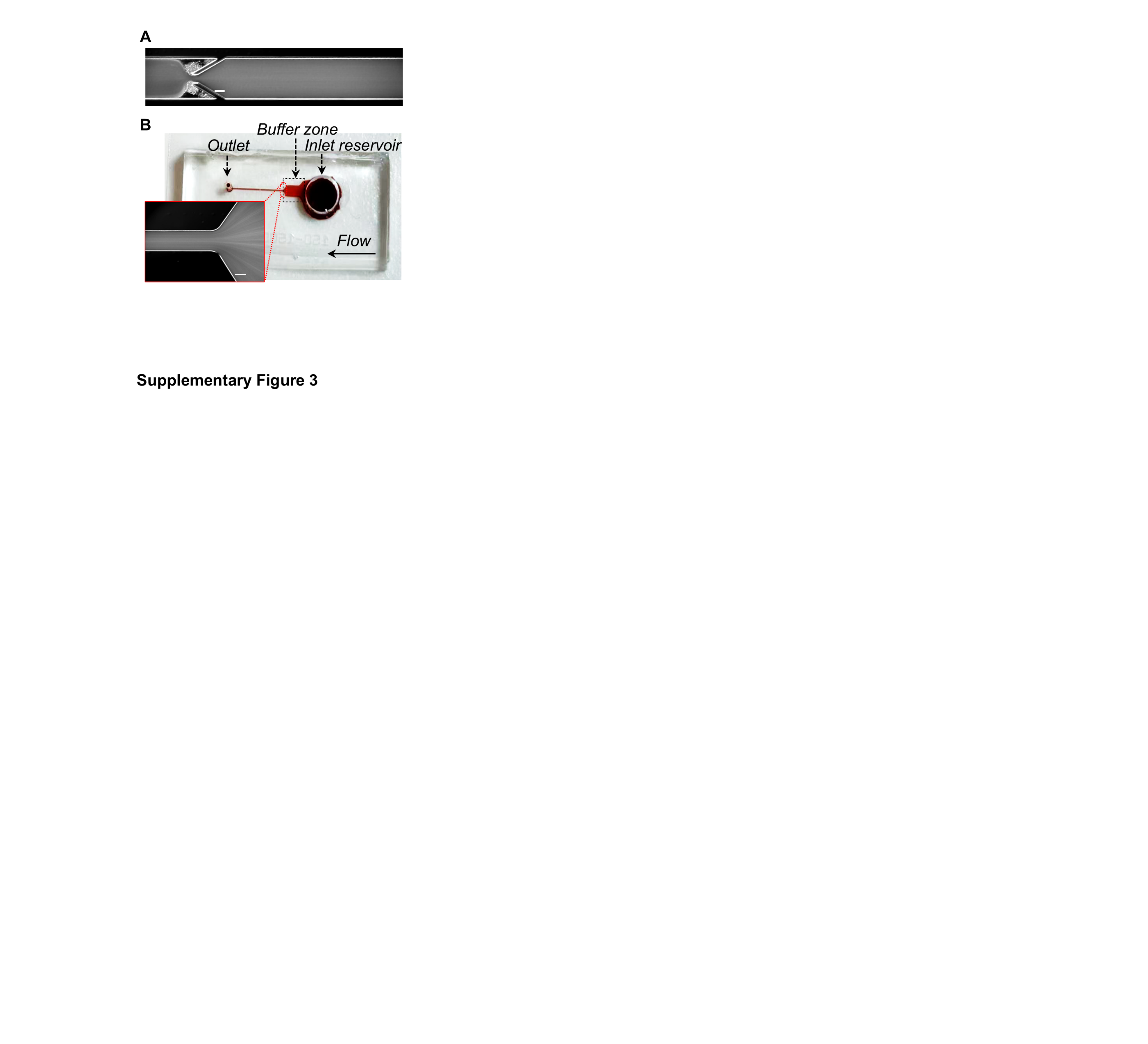


**Supplementary Figure S3: Confirmation of in situ thrombus formation in vortex regions.**

A) Representative DIC images after 300 seconds in vorticity microfluidic channels of β = 150° at 2,000 s^-1^ with 10× magnification objective, allowing visualization of approximately 1.5mm of the microfluidic channel. B) A top view photograph of the PDMS microfluidic device with inlet, outlet and flow direction indicated. The buffer zone was designed at the inlet, where minimizes shear stress variations. The connection between the straight channel and the buffer zone of the vorticity microfluidic channels is also highlighted. Thrombus formation begins primarily at the stenosis region and within the expansion zone where the vortex forms, rather than being initiated upstream and subsequently captured by the vortex. This confirms that vorticity directly influences local platelet activation and aggregation rather than merely capturing pre-formed thrombi. Scale bars: 50 µm (A) and 100 µm (B).


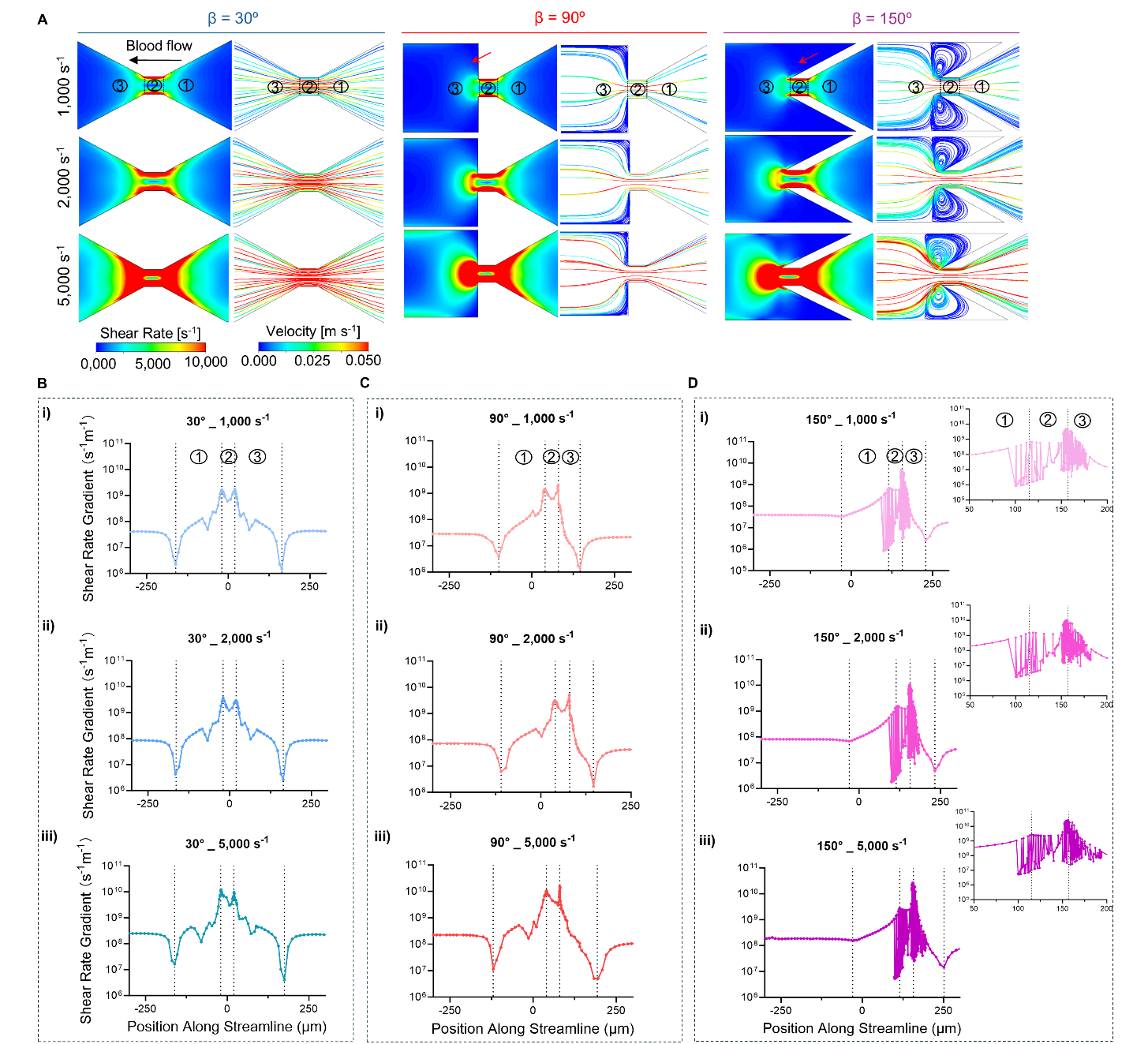


**Supplementary Figure S4**. CFD simulated shear rates and shear gradients at the mid-plane (z-axis) of the microfluidic channels at various input shear rates.

A) CFD simulated shear rate colormap (left) and velocity streamlines (right) of the centreplane of the microchannel for expansion angles with β = 30°, 90° and 150° under input shear rate of *γ_0_* = 1,000 (top), 2,000 (middle) and 5,000 (bottom) s^-1^ respectively. The interstitial blood flow rendering was colored by shear rate at constant viscosity. Note the shear rate maxima occurs at the stenosis apex and near the wall, where frictional forces are greatest, while the center forms a low shear pocket. Low shear pockets (red arrows) were observed in β = 90° and 150° after the stenosis area, and the area of it decreased with the flow increased. The microfluidic channels were divided into three zones based on the flow pattern: (1) Contraction zone, (2) Constriction zone and (3) Expansion zone. The blood flowing from the inlet ramps up accelerating within the constriction zone reaching a maximum shear rate in the stenosis apex, following this there is a deceleration in the blood flow velocity as microfluidic channel expands in the expansion zone. B-D) Shear rate gradient *γ’* values taken from the flow streamline that produced the largest vortical structure for β = 30° (B), 90° (C) and 150° (D) under input shear rates of *γ_0_* = 1,000 (top, Bi, Ci, Di), 2,000 (middle, Bii, Cii, Dii) and 5,000 s^-1^ (bottom, Biii, Ciii, Diii) respectively.


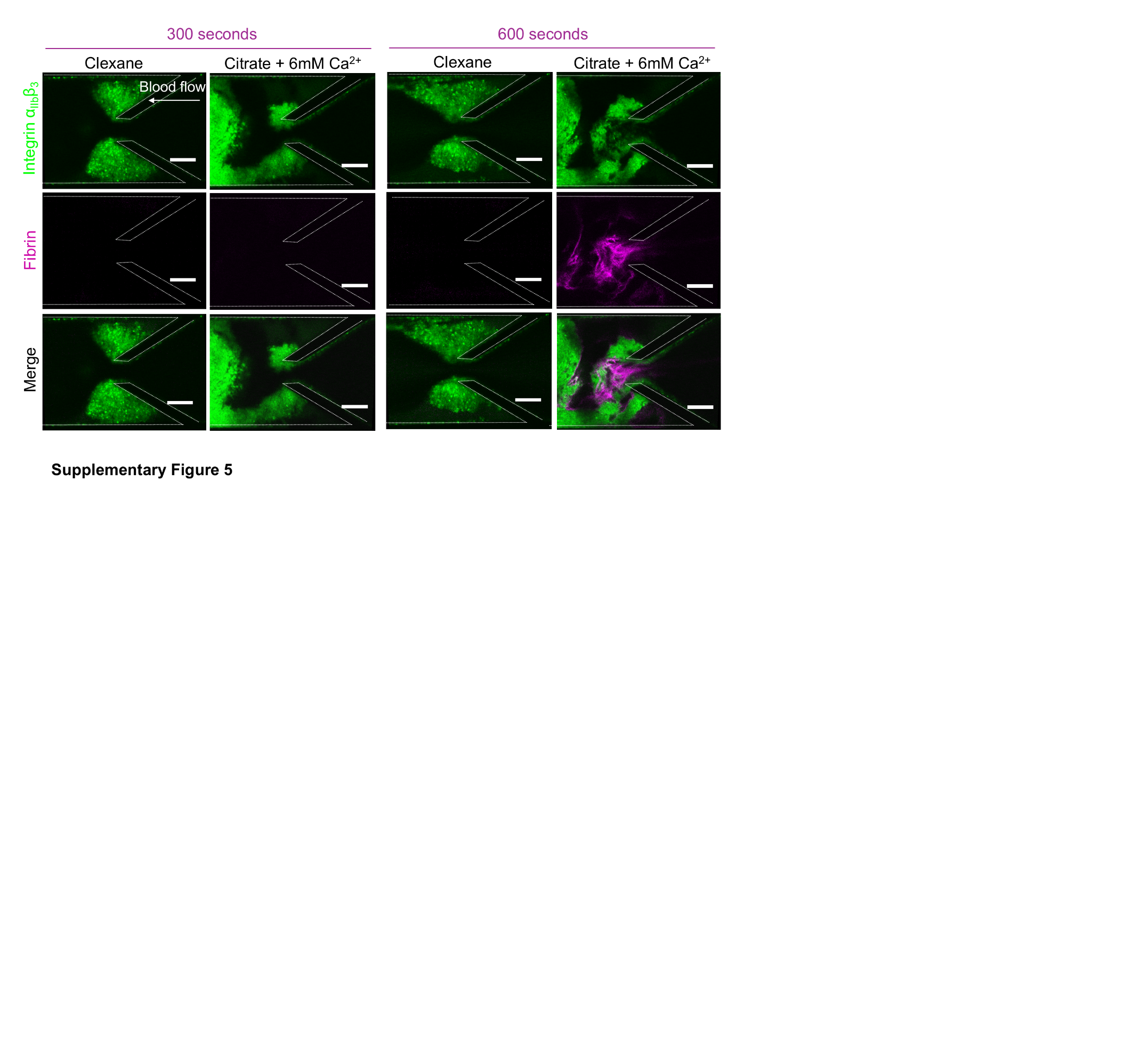


**Supplementary Figure S5: Absence of hypercoagulable state during platelet aggregation in vortex regions.**

Representative confocal images of platelet aggregation (anti-integrin α_IIb_β_3_ mAb P2,, *green*) and fibrin formation (*magenta*) in β = 150° after 300- and 600-second whole blood (WB) perfusion at *γ_0_* = 2,000 s^-1^. WB was treated with Clexane and 3.8% sodium citrate anticoagulant respectively, in which citrate blood was recalcified with 6mM final calcium concentration. The absence of fibrin signal within the vortex zone during the 300-second perfusion period confirms that coagulation activation does not contribute to vortex-related platelet aggregation. In contrast, the positive control using recalcified citrated blood shows fibrin signals beginning to appear after 300 seconds. This temporal separation demonstrates that the development of a hypercoagulable state requires a longer time frame than our experimental window, confirming that the thrombi observed in our experiments are formed through biomechanical platelet aggregation driven by vorticity and shear forces, rather than a consequence of blood stasis-induced hypercoagulability. Scale bars: 50 µm.

**Supplementary Videos**

**Supplementary Video S1. Whole-channel visualization of platelet dynamics during perfusion.**

Representative DIC video showing platelet behavior throughout the microfluidic channel of β = 150° geometry during 300-second perfusion at *γ_0_* = 2,000 s^-1^ shear rate, captured using a low magnification (10×) objective. The video demonstrates minimal platelet aggregation in the upstream region (from inlet to stenosis) throughout the entire perfusion period. The upstream inlet regions experience relatively low shear rates and are not conducive to significant platelet activation or aggregation, especially given the use of Clexane-anticoagulated blood. Thrombus formation is observed to begin primarily at the stenosis region and within the expansion zone where the vortex forms, rather than being initiated upstream. Individual platelets can be seen flowing through the channel without forming significant aggregates upstream but beginning to adhere and accumulate specifically in the vortex region. This provides direct evidence that the thrombi observed in the system are formed in situ within the vortex region rather than being transported from upstream locations, confirming that vorticity directly influences local platelet activation and aggregation. Scale bar: 50 µm.

**Supplementary Video S2. Vorticity-related rapid platelet accumulation under consistent shear conditions.**

Representative DIC videos for vorticity-related rapid platelet accumulation to the expansion zone under *γ_0_* = 2,000 s^-1^ in β = 30°, 90° and 150° geometries. The videos demonstrate the differences in platelet aggregation dynamics between low and high vorticity conditions. In the β = 30° geometry, platelet aggregation is primarily localized to the stenosis region with minimal accumulation in the expansion zone. As the expansion angle increases to β = 90° and especially β = 150°, progressively larger aggregates form in the expansion zone where vortices develop. Notably, in all geometries, thrombus formation initiates at the stenosis region and within the expansion zone, rather than being transported from upstream locations, confirming the direct influence of local flow conditions on platelet activation and aggregation. Scale bars: 50 µm.

**Supplementary Video S3. Wall shear rate modulation of vorticity-related platelet aggregation.**

Representative DIC videos for wall shear rate modulation of vorticity-related platelet aggregation in β = 150° under *γ_0_* = 1,000 s^-1^, 2,000 s^-1^ and 5000 s^-1^. The videos illustrate the synergistic effect between high shear rates and strong vorticity in promoting platelet aggregation. At the lowest shear rate (*γ_0_* = 1,000 s^-1^), platelet aggregation in the vortex region develops gradually over the 300-second observation period. As the shear rate increases to *γ_0_* = 2,000 s^-1^ and further to 5,000 s^-1^, the rate of platelet accumulation rises sharply, leading to rapid and extensive thrombus formation within the vortex region during the first 3 minutes of perfusion. At *γ_0_* = 5,000 s⁻¹, thrombus growth not only initiates quickly but also continues progressively throughout the entire observation period, indicating a strong correlation between high shear conditions and sustained platelet-driven thrombus development. These findings highlight how extreme flow conditions, combining high shear stress with strong vorticity, create ideal conditions for accelerated thrombus formation, mimicking conditions often found in medical devices associated with thrombotic complications. Scale bars: 50 µm.
